# Two shikimate dehydrogenases, *VvSDH3* and *VvSDH4*, are involved in gallic acid biosynthesis in grapevine

**DOI:** 10.1093/jxb/erw184

**Published:** 2016-05-28

**Authors:** Thibaut Bontpart, Thérèse Marlin, Sandrine Vialet, Jean-Luc Guiraud, Lucie Pinasseau, Emmanuelle Meudec, Nicolas Sommerer, Véronique Cheynier, Nancy Terrier

**Affiliations:** INRA, UMR 1083 Sciences pour l’œnologie, 2 place Pierre Viala, F-34060 Montpellier cedex 1, France

**Keywords:** Flavan-3-ol, flavonoid, gallic acid, galloylation, grapevine, shikimate dehydrogenase.

## Abstract

In plants, the shikimate pathway provides aromatic amino acids that are used to generate numerous secondary metabolites, including phenolic compounds. In this pathway, shikimate dehydrogenases (SDH) ‘classically’ catalyse the reversible dehydrogenation of 3-dehydroshikimate to shikimate. The capacity of SDH to produce gallic acid from shikimate pathway metabolites has not been studied in depth. In grapevine berries, gallic acid mainly accumulates as galloylated flavan-3-ols. The four grapevine SDH proteins have been produced in *Escherichia coli*. *In vitro*, VvSDH1 exhibited the highest ‘classical’ SDH activity. Two genes, *VvSDH3* and *VvSDH4*, mainly expressed in immature berry tissues in which galloylated flavan-3-ols are accumulated, encoded enzymes with lower ‘classical’ activity but were able to produce gallic acid *in vitro*. The over-expression of *VvSDH3* in hairy-roots increased the content of aromatic amino acids and hydroxycinnamates, but had little or no effect on molecules more distant from the shikimate pathway (stilbenoids and flavan-3-ols). In parallel, the contents of gallic acid, β-glucogallin, and galloylated flavan-3-ols were increased, attesting to the influence of this gene on gallic acid metabolism. Phylogenetic analysis from dicotyledon SDHs opens the way for the examination of genes from other plants which accumulate gallic acid-based metabolites.

## Highlight

Functional analysis of the four grapevine shikimate dehydrogenases (*VvSDH1–4*) reveals that two of them are involved in gallic acid biosynthesis.

## Introduction

Grapevine (*Vitis vinifera* L.) is one of the most widespread fruit plants in the world. Mainly cultivated for winemaking, it is a crop of economic interest found in numerous world regions with a temperate climate. Grape berry growth is characterized by a double sigmoid curve (Coombe and Hale, 1973). Véraison marks the beginning of the ripening stage. Proanthocyanidins (PAs) are oligomers and polymers of flavan-3-ols. They are the major flavonoids in grape berry and wine and influence astringency, bitterness, and colour stability (Arnold *et al.*, 1980). Their biosynthesis starts before flowering and their accumulation peak occurs around véraison in seeds and skin (Bogs *et al.*, 2005). The main PA subunit, (-)-epicatechin, is partly acylated with gallic acid (GA), a trihydroxybenzoic acid, to form (-)-epicatechin 3-*O*-gallate. The percentage of (-)-epicatechin 3-*O*-gallate in a PA chain defines the galloylation rate (%G). %G is higher in seeds than in skin and pulp (Cheynier, 2005; Verriès *et al.*, 2008). Galloylation increases the antiproliferative (Lizarraga *et al.*, 2007), oenological (Vidal *et al.*, 2003), and colloidal (Fabre *et al.*, 2010) properties of PAs. The GA glucose ester, named β-glucogallin (β-G), is a precursor for the biosynthesis of both hydrolysable tannins (Haslam and Cai, 1994; Niemetz and Gross, 2005) and galloylated PAs (Liu *et al.*, 2012).

**Fig. 1.**
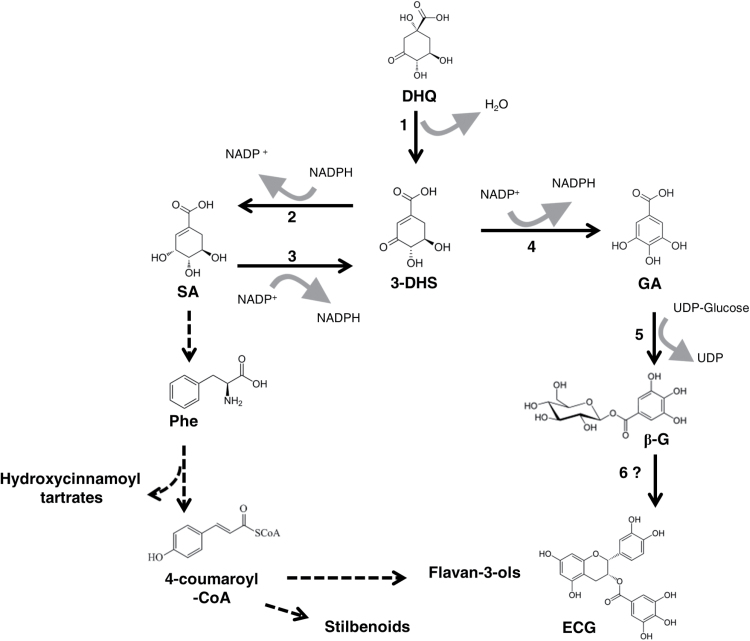
Gallic and shikimic acid biosynthesis in plants. Reaction 1: Dehydroquinic acid (DHQ) dehydration catalysed by the dehydroquinate dehydratase (DQD) domain of the dehydroquinate dehydratase/shikimate dehydrogenase (DQD/SDH) producing 3-dehydroshikimic acid (3-DHS). Reaction 2: NADPH-dependent reduction of 3-DHS catalysed by the shikimate dehydrogenase (SDH) domain of DQD/SDH producing shikimic acid (SA). Reaction 3: NADP^+^-dependent oxidation of SA catalysed by the SDH domain of DQD/SDH producing 3-DHS. Reaction 4: NADP^+^-dependent oxidation of 3-DHS catalysed by the SDH domain of DQD/SDH producing gallic acid (GA). Reaction 5: UDP-Glucose-dependent glucosylation of gallic acid catalysed by grapevine glucosyltransferases (Khater *et al.*, 2012). Reaction 6?: Putative galloylation of epicatechin from β-glucogallin. Dotted lines indicate pathways of several steps.

GA biosynthesis has been studied since the 1960s (Dewick and Haslam, 1969). A retrobiosynthetic NMR study with labelled glucose supplied to the young leaves of Sumac highlighted that the carboxylic group of GA comes from an intermediate of the shikimate (SA) pathway (Werner *et al.*, 1997). The SA pathway is known to provide carbon structures used for the biosynthesis of aromatic amino acids and downstream of different secondary metabolites. However, branches from this pathway would also lead to (hydroxy)benzoic acids (Widhalm and Dudareva, 2015). From crude extracts of birch leaves (*Betula pubescens* ssp. *Czerepanovii*), GA biosynthesis from 3-dehydroshikimate (3-DHS) and NADP^+^ has been reported (Fig. 1, reaction 4; Ossipov *et al.*, 2003). Recently, it has also been suggested that, in addition to its already known catalytic properties, an SDH could be responsible for GA biosynthesis in walnut (*Juglans regia*; Muir *et al.*, 2011). However, GA biosynthesis in plants has not been investigated beyond these studies. The dehydroquinate dehydratase/shikimate dehydrogenase (DQD/SDH, EC 4.2.1.10/1.1.1.25) is known as a bifunctional enzyme catalysing the third and the fourth steps of this pathway (Maeda and Dudareva, 2012): the dehydration of 3-dehydroquinate and the NADPH-dependent reduction of 3-DHS (Fig. 1, reactions 1 and 2, respectively). The latter reaction is reversible: SA can be subject to a NADP^+^-dependent oxidation to form 3-DHS (reaction 3).

Transcriptomic analyses of grapevine hairy-roots over-expressing transcription factors controlling PA biosynthesis has established a list of induced genes that are potentially involved in PA biosynthesis (Terrier *et al.*, 2009). A Quantitative Trait Loci (QTL) analysis has been performed to locate the loci of genes able to influence the different characteristics of PAs, notably %G (Huang *et al.*, 2012). By an integrative approach focusing on PA concentration and %G, a list of 20 candidate genes was established (Carrier *et al.*, 2013) including a gene encoding a DQD/SDH, here referred to as *VvSDH4*. This gene is far upstream of the flavonoid pathway and we hypothesized that it could be involved in GA biosynthesis, thus indirectly affecting the galloylation rate of flavan-3-ols. Using the same screening approach, three grapevine UDP-glucosyltransferases (VvgGTs) were also identified. They are expressed in the early stages of grape berry development and could catalyse the biosynthesis of β-G, as demonstrated *in vitro* (Fig. 1, reaction 5) by Khater *et al.* (2012). Several studies suggest that Serine Carboxypeptidase-Like (SCPL) acyltransferase(s) could be involved in the further step of flavan-3-ol galloylation, using β-G (for a review see Bontpart *et al.*, 2015; Fig. 1, reaction 6).

*VvSDH4* is one of the four genes encoding DQD/SDH in the grapevine genome. A gene (designated *VvSDH1*) is located on chromosome 5. The other three genes (designated *VvSDH2*, *VvSDH3*, and *VvSDH4*) are located as a cluster on chromosome 14, as identified by a QTL analysis of %G (see Supplementary Fig. S1 at *JXB* online, Carrier *et al.*, 2013).

In the present study, we have cloned and sequenced the four genes annotated as DQD/SDH which are carried by the grapevine genome in order to test their capacity to produce GA. Heterologous expression of the encoded proteins was performed in *Escherichia coli* (*E. coli*). Kinetic parameters (substrate and cofactor affinity, pH effect) have been determined *in vitro* for SA oxidation and 3-DHS reduction (Fig. 1, reactions 3 and 2, respectively) for each VvSDH. *In vitro* GA production from both substrates has been evaluated. *In vivo*, the content of aromatic amino acids and phenolic compounds derived from the SA pathway has been quantified in grapevine hairy-roots over-expressing *VvSDH3*.

## Materials and methods

### Plant material

All grapevine material was harvested in the INRA-Supagro vineyard (Montpellier, France). Grape berries (*Vitis vinifera* L. cv. Syrah) were harvested at different development stages and classified according to elapsed time from anthesis: 11, 18, 35, 52, 56, 58, 65, 86, and 99 d after flowering (daf). Fifty-two days after flowering marks the start of fruit ripening (véraison). Skin, pulp, and seeds of berries harvested at 18 daf (green stage), 52 daf (véraison), and 99 daf (maturity) were isolated. Immediately after harvest, the berries were frozen in liquid nitrogen. The fruits were ground using a Dangoumau blender (Dangoumill 300, Lonjumeau, France) and stored at –80 °C.

Grapevine vitro-plants (cv. Maccabeu), used to generate transgenic hairy-roots, were grown in a culture chamber (24 °C, 15h photoperiod).

### RNA extraction and quantitative real-time PCR

Extraction of total RNA from 100mg of powder was performed using the RNeasy Plant Mini kit (Qiagen, Hilden, Germany). The concentration of total RNA isolated from berries was quantified with Nanodrop 3300 (ThermoScientific, Wilmington, USA). One µg of isolated RNA was used for reverse transcription using ImProm-II^TM^ Reverse Transcription System (Promega) in a total volume of 20 µl. One µl of synthesized cDNA was amplified in triplicate by quantitative real time PCR using specific primers of *SDH* genes (Supplementary Table S1) and the Power SYBER-Green PCR Master-kit (Applied Biosystems, Applera France, Courtaboeuf, France). PCR was carried out with the 7 300 Real-Time PCR System (Applied Biosystems) and analysed with 7 300 System SDS Software v 1.3.1.

To normalize *VvSDH* expression, Elongation factor 1α (*EF1α*) was used as the reference gene (Monteiro *et al.*, 2013). Relative *SDH* expression was calculated from the cycle threshold (Ct) according to formula 2 exp-(Ct_*SDH*_–Ct_*EF1α*_).

### Cloning and vectors

*VvSDH* cDNA was amplified from *Vitis vinifera* L. berries cv. Syrah collected at the green stage with high fidelity polymerase (Advantage–HF 2 PCR kit, Clontech, California, USA). The forward and reverse primers for cloning into pGEMT-Easy (Promega, Madison, Wisconsin, USA) were designed to incorporate the restriction site for *Bam*HI before the start codon (except for *VvSDH4*) and *No*tI after the stop codon, respectively (Supplementary Table S1). A partial cDNA of *VvSDH4* was cloned into pGEX-4T2 to produce the protein used in this study. This lacks the apparent transit peptide composed of the first 17 amino acids in the N-terminal region as described for NtSDH1 (Ding *et al.*, 2007), to avoid the formation of inclusion bodies. The purified insert was cloned into pGEMT-Easy and digested with the same restriction enzymes. The DNA fragments obtained were cloned into pGEX-4T2 previously digested with the same restriction enzymes (GE Healthcare, Chalfont St Giles, UK).

Full-length cDNA from *VvSDH3* and *VvSDH4* were cloned in parallel into the pENTR/D-TOPO vector (Invitrogen, Courtaboeuf, France) and transferred in the pH2GW7 vector (Karimi *et al.*, 2002) by the LR reaction (LR clonase II, Invitrogen). The complete sequence of *VvSDH4* cloned in pH2GW7 was used for sequence analysis. Competent cells of *E. coli* DH5α were used to incorporate and propagate all vectors, except pGEX-4T2 for which BL21 (DE3) cells (Stratagene, La Jolla, California, USA) were used.

The Genbank accession numbers corresponding to full length nucleotide sequences are KU163040 (*VvSDH1*), KU163041 (*VvSDH2*), KU163042 (*VvSDH3*), and KU163043 (*VvSDH4*).

### Phylogenetic analysis

Protein sequences of plant DQD/SDHs were collected from the NCBI public database and Phytozome 10.0. Phylogenetic analyses were performed using MEGA version 6 (Tamura *et al.*, 2013). Protein sequences were aligned using ClustalW with a gap open penalty of 10, and a gap extension penalty of 0.2. A Neighbor–Joining tree with the evolutionary distances was computed using the p-distance model. The statistical support for tree nodes was evaluated by the Bootstrap method (1 000 bootstrap replicates). The presence of a putative chloroplastic transit peptide has been predicted using ChloroP 1.1 (Emanuelsson *et al.*, 2006).

### Heterologous expression in *E. coli*

Heterologous expression, purification, and quantification of VvSDH were performed as described in Khater *et al.* (2012). Purity and molecular weight of the recombinant proteins were checked by running an aliquot on SDS-PAGE (14%) by electrophoresis (data not shown). Recombinant proteins were diluted in glycerol 16% (v/v), divided into aliquots, and stored at –20 °C.

### Measuring the activity of recombinant proteins

For kinetic parameter measurements, VvSDH activity using SA as the substrate and NADP^+^ as the cofactor (reaction 3, Fig. 1) was determined by monitoring NADPH variation for 2min at OD_340_ in UV-cuvettes using a spectrophotometer (Cary 100 Bio UV-Visible, Varian). The same method was used to measure VvSDH activity from 3-DHS as the substrate and NADPH as the cofactor (reaction 2, Fig. 1).

All reactions were performed at 30 °C in a final volume of 200 µl containing 100mM Bis-Tris Propane (BTP) HCl buffer and started by adding 100ng of enzyme to the reaction mixture. No OD_340_ variation was observed without the enzyme.

The effect of pH was assayed with 10mM of substrate and 2mM of cofactor using 100ng of recombinant proteins in 100mM BTP buffer. The optimal pH for each enzyme has been determined using a buffer with a pH range from 6.5 to 9.

*K*_m_ for SA (*K*_m (SA)_) was determined at pH 7 and pH 9, with reaction mixtures containing 2mM NADP^+^ and variable SA concentrations. *K*_m (NADP+)_ was determined at the same pH with 4mM SA and variable NADP^+^ concentrations. *K*_m (3-DHS)_ was determined at pH 7 for VvSDH1 and VvSDH3 using 4mM 3-DHS and NADPH as the cofactor (0.4mM).

For each substrate or cofactor concentration, OD_340_ was plotted as a function of time to determine the slope at the origin. Initial velocity (*V*_i_) was calculated with the NADPH molar extinction coefficient (ε=6.18×10^3^ mol l^−1^). *V*_i_ data was fitted with Michaelis–Menten hyperbola to yield *K*_m_ and *V*_max_ values (Hyper32 program, Version 1.0.0, 2003). The turnover number (*k*_cat_) was calculated, taking into account the quantity of enzyme introduced in the reaction volume. The catalytic efficiency was calculated as *k*_cat_/*K*_m_.

### Analysis and quantification of enzyme products

For 3-DHS and GA quantification, 1 µg of SDH was added to a reaction mixture containing 100mM BTP buffer pH 9, 4mM SA or 3-DHS, 2mM NADP^+^, and 8mM ascorbic acid in a final volume of 80 µl. After 2h of incubation at 30 °C, the reaction was stopped with 20 µl HCl (final concentration 2.3M).

Five µl of the reaction mixture were injected into an Agilent 1100 LC system (Agilent Technologies, Waldbronn, Germany) equipped with a diode-array detector (DAD). Compounds were separated using an Atlantis dc18 (250×2.1, 5 µm) analytical column (Waters, Milford, MA) protected by an Atlantis dc18 (10×2.1, 5 µm) precolumn (Waters, Milford, MA).

The following method was developed to separate SA, 3-DHS, NADP^+^, GA, and protocatechuic acid (PCA) and to quantify 3-DHS and GA using UV-DAD (λ=280nm). The identification of molecules was achieved by a comparison of retention times and UV–visible spectra with those of authentic standards (Sigma-Aldrich).

The mobile phase consisted of water/formic acid (99.9/0.1, v/v) (eluent A) and methanol/formic acid containing 0.5% formic acid (99.5/0.5, v/v) (eluent B).

The elution programme had a 0.25ml min^–1^ flow rate and started with 0% B. The linear gradients were from 0–40% B (0–11min), from 40–50% B (11–15min), and from 50–100% B (15–25min), followed by a column reconditioning time. This programme was performed at 30 °C.

Mass spectra of 3-DHS and GA in enzymatic assays were achieved by Ultra Performance Liquid Chromatography coupled to Diode Array Detection and Mass spectrometry (UPLC-DAD-MS). Separations were performed using a Waters Acquity UPLC-DAD system (Milford, MA), on a (15×1mm i.d.) Acquity BEH C18 column (Waters, Milford, MA; 1.7 μm), operated at 35 °C. The mobile phase consisted of water/formic acid (99/1, v/v) (eluent A) and methanol/formic acid (99/1, v/v) (eluent B). The flow rate was 0.08ml min^−1^.

The elution programme was as follows: isocratic for 4min with 1% B, 1–20% B (4–10min), 20–98% B (10–12min), isocratic with 98% B (12–17min).

ESI-MS/MS analyses were performed with a Bruker Daltonics Amazon (Bremen, Germany) mass spectrometer equipped with an electrospray source and an ion trap mass analyser. The spectrometer was operated in the positive and negative ion modes (end plate offset: –500V; temperature, 200 °C; nebulizer gas: 10 psi and dry gas, 5 l min^−1^; capillary voltage, 2.5kV in the positive ion mode, 4.5kV in the negative ion mode). Collision energy for fragmentation used for MS2 experiments was set at 1.

### Grapevine hairy-root generation

Induction and culture of hairy-roots was monitored as described in Gomez *et al.* (2009). After several subcultures, hairy-roots were extracted from agar medium, cleaned, weighed, and frozen in liquid nitrogen. The samples were cold-milled using a 6770 Freezer/Mill^®^ cryogenic mill (SPEX^®^ SamplePrep, USA) into a fine powder. The powder was used for DNA and RNA extraction and the analysis of metabolites. Hairy-roots devoid of transgene were used as a control.

### Plant secondary metabolite profiling

Approximately 100mg of frozen powdered grapevine hairy-root or berry tissues were weighed and transferred into a Precellys^®^ tube (7ml) containing beads. 500 µl of pure methanol was added directly and mixed by vortexing in order to inhibit oxidation. Three and a half millilitres of acetone:water:trifluoroacetic acid (70:30:0.05 by vol.) were then added to the extract. The sample was ground using a grinder-homogenizer (Precellys24^®^) with a cycle of three pulses of 40s at 5 000rpm with 40s between each pulse. This cycle was repeated three times. The homogenate was transferred to a 15ml tube and centrifuged (5min, 4 500rpm, 4 °C). Two fractions of 1ml of supernatant were then dried (Genevac, SP Scientific).

The pellet of one of the two fractions was resuspended in 500 µl of methanol:water:formic acid (50:50:1, by vol.) and placed in an ultrasonic bath. After 30min, the sample was centrifuged (15min, 15 000rpm, 4 °C) and the supernatant was recovered for analysis.

The other fraction underwent a phloroglucinolysis step before injection. The phloroglucinolysis reagent was freshly prepared with 0.25g of phloroglucinol and 0.05g of ascorbic acid with 5ml of acidified methanol (final HCl concentration: 0.2M). The pellet used for phloroglucinolysis was resuspended in 700 µl of phloroglucinolysis reagent using a sterile needle. After vortexing, the sample was placed in a water bath at 50 °C for PA depolymerization. After 20min of incubation, the tube was cooled on ice and 700 µl of ammonium formate buffer 200mM was added to stop the reaction. After centrifugation (15min, 15 000rpm, 4 °C), the supernatant was recovered and filtered before analysis.

Hairy-roots and grape berry metabolites were analysed using Ultra High Performance Liquid Chromatography coupled to triple-quadrupole Mass Spectrometry (UHPLC-QqQ-MS) following a method adapted from that described by Lambert *et al.* (2015). This method has been used for the quantification of metabolites after direct injection, and depolymerized PA subunits after depolymerization by phloroglucinolysis. β-G was a kind gift of Dr Gross (retired, formerly University of Ulm, Germany).

Flavan-3-ols from grape berry tissues have been analysed as described by Verriès *et al.* (2008).

### Aromatic amino acid analysis

Free and structural aromatic amino acids were assayed separately with an amino acid analyser (Biochrom 30, Biochrom) from the frozen powder of hairy-roots.

The free form was obtained by extraction for 1h with 0.2M lithium citrate, sulphosalicylic acid 5% (w/v), 4 °C, and shaking, followed by a higher molecular weight molecule precipitation step for 1h at 4 °C on ice, and a final centrifugation for 15min at 15 000rpm and 4 °C.

The structural amino acid fraction was assayed after biomass hydrolysis (24h incubation at 110 °C in the presence of 6M HCl).

In both cases, the sample was filtered through a 0.22 μm pore-sized nitrocellulose membrane filter (Millipore). Amino acids were separated by liquid chromatography on an ion-exchange column (Ultrapac-8 Lithium form; Amersham Pharmacia Biotech), and were detected by a post column reaction with ninhydrin reagent. Concentrations were determined from peak areas at 570nm and compared with the injection of corresponding commercial standards (Sigma-Aldrich). Norleucine (0.5mM) was added to the samples as an internal standard.

### Statistical analysis

The significance of the results from HPLC and UHPLC-QqQ-MS analysis has been statistically assessed with a Student’s *t* test using two-sided alternative with the R software (version 3.12, R Development Core Team, 2009).

## Results

### Identification of four DQD/SDHs

According to the 12X version of the *Vitis vinifera* genome and the V2 annotation (http://genomes.cribi.unipd.it/grape/), *VvSDH1* is located on chromosome 5 whereas the other three genes are clustered on chromosome 14 (Supplementary Fig. S1). This cluster is 34.149kb long. The genomic DNAs of VIT_205s0020g02030 (*VvSDH1*), VIT_214s0030g00670 (*VvSDH2*), VIT_214s0030g00660 (*VvSDH3*), and VIT_214s0030g00650 (*VvSDH4*) are 32.141, 9.695, 4.23, and 9.573kb long, respectively. The exon–intron pattern corresponding to the cloned cDNAs is shown in Supplementary Fig. S1. *VvSDH1*, *VvSDH2*, *VvSDH3*, and *VvSDH4* full-length cDNAs are 1560, 1560, 1596, and 1596 nucleotides long, encoding proteins constituted of 520, 520, 532, and 532 amino acids with predicted molecular weights of 56.54, 57.17, 57.68, and 57.28kDa, respectively (ExPASy server, Gasteiger *et al.*, 2003). Full-length cDNAs were used for sequence alignment and phylogenetic analysis (Supplementary Alignment S1). VvSDH1, VvSDH3, and VvSDH4 share more than 70% of the nucleotide and amino acid identity. VvSDH2 shares less than 60% of the nucleotide identity and about 50% of amino acids identity with the other three sequences (Supplementary Table S2).

### Phylogenetic analysis

We have tested whether DQD/SDHs from different species across the dicotyledons could share high sequence identity. The scientific and common name, the plant family, and the number of protein sequences analysed are listed in Supplementary Table S3.

The Neighbor–Joining tree has been constructed with VvSDH sequences and previously characterized DQD/SDHs from *Arabidopsis thaliana* (*At*, Singh and Christendat, 2006), *Populus trichocarpa* (*Poptr*, Guo *et al.*, 2014), *Juglans regia* (*Jr*, Muir *et al.*, 2011), and *Nicotiana tabacum* (*Nt*, Ding *et al.*, 2007). Considering that GA takes part in tannin metabolism, we have included DQD/SDH sequences of plants known to accumulate high amount of galloylated flavan-3-ols: *Camellia sinensis* (Cas, Jiang *et al.*, 2013), *Diospyros kaki* (*Dk*, Ikegami *et al.*, 2005), and/or ellagitannins, that are polyphenols formed from the oxidative linkage of galloyl groups from pentagalloyl glucose, such as *Fragaria vesca* (*Fv*, Landete, 2011) and *Eucalyptus* sp. (*Eg*, Boulekbache-Makhlouf *et al.*, 2010). We also chose two other plants whose genome has been entirely sequenced, *Solanum lycopersicum* (*Sl*) and *Citrus sinensis* (*Cs*), to collect an exhaustive list of DQD/SDH sequences from other dicots.

**Fig. 2.**
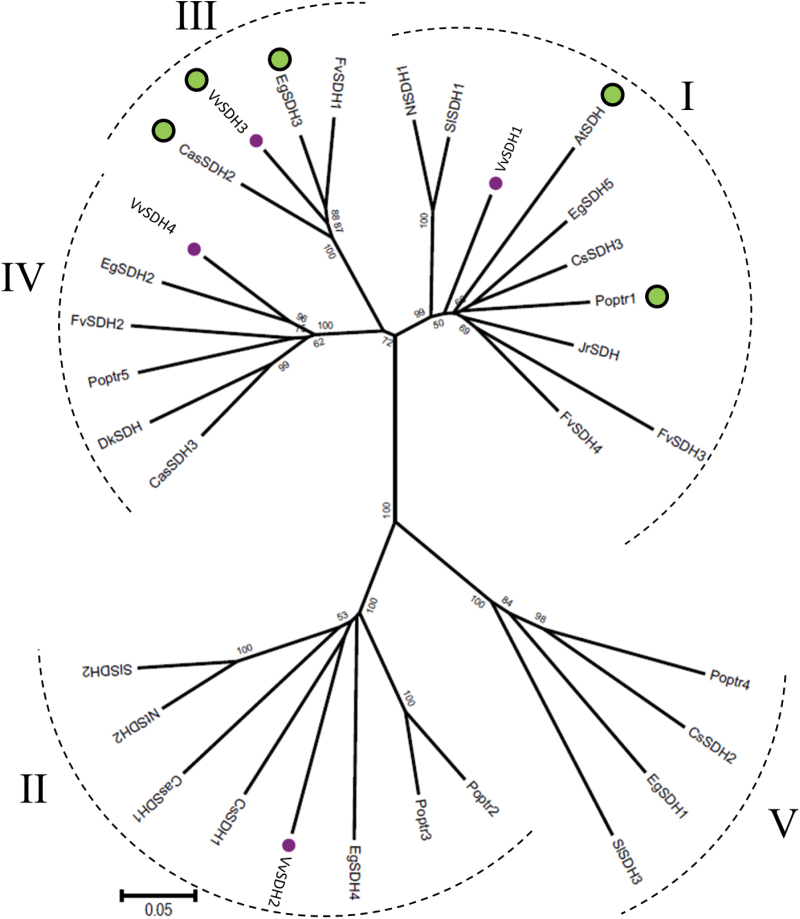
Neighbor–Joining tree of selected dehydroquinate dehydratase/shikimate dehydrogenases from dicots. This phylogenetic tree was constructed from the four VvSDHs sequenced in this study and the 28 sequences available on public databases (NCBI and Phytozome) using MEGA6 software. Bootstrap values (percentage of 1 000 replicates) are shown for key branches. The scale bar represents 0.05 substitutions per site. Abbreviations: At (*Arabidopsis thaliana*), Cs (*Citrus sinensis*), Cas (*Camellia sinensis*), Dk (*Diospyros kaki*), Eg (*Eucalyptus grandis*), Fv (*Fragaria vesca*), Jr (*Juglans regia*), Nt (*Nicotiana tabacum*), Poptr (*Populus trichocarpa*), Sl (*Solanum lycopersicum*), Vv (*Vitis vinifera*). Accession numbers: Group I: AtSDH (AAF08579), FvSDH3 (XP_004289250), FvSDH4 (XP_004288087), VvSDH1 (KU163040), EgSDH5 (Eucgr.J00263.6), NtSDH1 (AAS90325), Poptr1 (Potri.010G019000), SlSDH1 (AAC17991), CsSDH3 (orange1.1g007151m), JrSHD (AAW65140); Group II: CasSDH1 (AIZ93902), CsSDH1 (orange1.1g010050m), NtSDH2 (AAS90324), VvSDH2 (KU163041), EgSDH4 (Eucgr.B01770.2), Poptr2 (Potri.013G029900), Poptr3 (Potri.005G043400), SlSDH2 (XP_010327280); Group III: EgSDH3 (Eucgr.H04427.1), FvSDH1 (XP_004302480), VvSDH3 (KU163042), CasSDH2 (AJA40947); Group IV: EgSDH2 (Eucgr.H04428.1), VvSDH4 (KU163043), Poptr5 (Potri.013G029800), FvSDH2 (XP_004302479), DkSDH (BAI40147), CasSDH3 (AJA40948); Group V: Poptr4 (Potri.014G135500), EgSDH1 (Eucgr.H01214.1), CsSDH2 (orange1.1g010101m), SlSDH3 (XP_004242317). Accession number corresponds to the NCBI or Phytozome 10.1.2 database. Purple dots show VvSDHs. The presence of a putative chloroplastic target peptide according to ChloroP is shown by a green dot.

We observed that DQD/SDHs, independently of their species of origin, cluster into five groups (I–V, Fig. 2).

The ten proteins constituting group I share about 75% sequence identity on average. VvSDH1 clusters with four characterized DQD/SDHs: AtSDH (Singh and Christendat, 2006), Poptr1 (Guo *et al.*, 2014), JrSDH (Muir *et al.*, 2011), and NtSDH1 (Ding *et al.*, 2007).

In group II, eight DQD/SDHs proteins share 71% sequence identity on average. In the proposed phylogenetic tree, VvSDH2 clusters with Poptr2 and Poptr3, two characterized DQD/SDHs (Guo *et al.*, 2014) and NtSDH2 (Ding *et al.*, 2007).

In group III, four DQD/SDHs exhibit about 85% sequence identity on average, representing the higher value among the four groups identified. It is interesting that this value is reached as the sequences belong to four different species (VvSDH3, CasSDH2, FvSDH1, and EgSDH3) which accumulate GA-based tannins.

Group IV consists of six DQD/SDHs belonging to six different species and sharing 77% sequence identity on average. VvSDH4 clusters with Poptr5, a DQD/SDH already characterized in poplar.

Four proteins sharing 68% sequence identity on average constitute group V. None of these proteins have been characterized yet.

A putative chloroplastic transit peptide has been detected using ChloroP 1.1 for some members of groups I (AtSDH, Poptr1, EgSDH1) and III (CasSDH2, VvSDH3, EgSDH3) (Fig. 2).

### Comparison of key amino acids for SDH activity

**Fig. 3.**
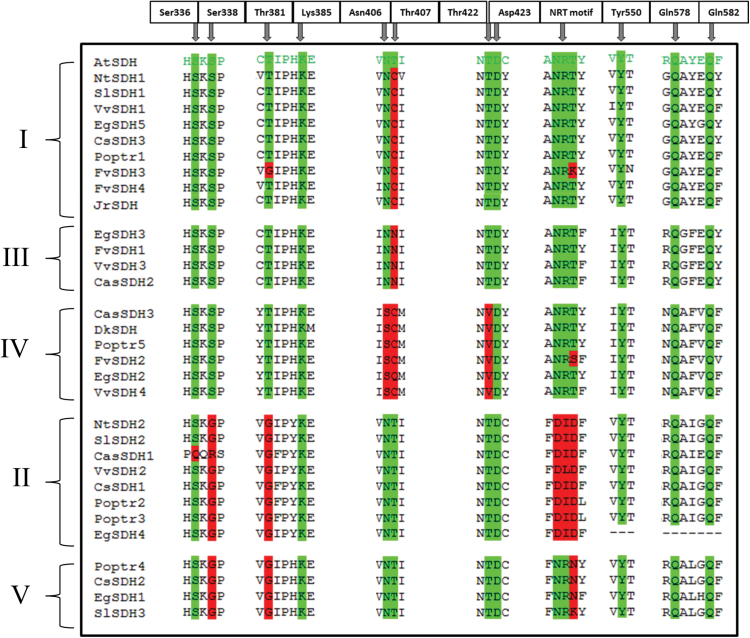
Comparison of key amino acids in the SDH domain of DQD/SDHs from dicots. Protein alignment has been performed by Clustal Omega. Amino acids numerotation is based on the DQD/SDH from *Arabidopsis thaliana* (AtSDH; Singh and Christendat, 2006). The key amino acids are shaded in green when they are identical to AtSDH, and in red when they are different from AtSDH. Groups I–V have been determined from the Neighbor–Joining tree. This sequence alignment includes DQD/SDHs from *Arabidopsis thaliana* (AtSDH), *Nicotiana tabacum* (NtSDH1 and 2), *Solanum lycopersicum* (SlSDH1–3), *Vitis vinifera* (VvSDH1–4), *Eucalyptus grandis* (EgSDH1–5), *Citrus sinensis* (CsSDH1–3), *Populus trichocarpa* (Poptr1–5), *Fragaria vesca* (FvSDH1–4), *Juglans regia* (JrSDH), *Camellia sinensis* (CasSDH1–3), *Diospyros kaki* (DkSDH).

Key amino acids for both DQD and SDH activities have been highlighted from the crystal structure of AtDQD/SDH (Singh and Christendat, 2006). The SDH domain ensures the reversible dehydrogenation of 3-DHS to produce SA. The DQD/SDH sequences collected have been aligned with AtDQD/SDH to examine the identity of key amino acids in the SDH domain (Fig. 3).

Amino acid divergences within the SDH domain, including the sequence between Ile 328 and Gly 588 of AtDQD/SDH, have been identified (Fig. 3). According to Singh and Christendat (2006), Ser 336, Ser 338, and Tyr 550 bind the C1 carboxylate in a trigonal arrangement. Even if Ser 336 and Tyr 550 are well conserved among all sequences, Ser 338 is replaced by a Gly in DQD/SDHs clustering in groups II and V (except that there is an Arg for CasSDH1). The C4 hydroxyl group of the substrate is bound by Asn 406 and the C5 hydroxyl group by Gln 578 and Gln 582. Thr 407 and Thr 422 are involved in substrate orientation. Both Gln residues are fully conserved among the DQD/SDHs sequences considered. However, Asn 406 is replaced by a Ser in group IV. Thr 407 is replaced by a Cys in groups I (except AtSDH) and IV (except EgSDH2), and by an Asn in group III. Moreover, Thr 422 is replaced by a Val in group IV. Lys 385 and Asp 423 were expected to be catalytic residues due to their proximity with the C3 hydroxyl, the site of deprotonation. Those two amino acids are fully conserved among the analysed sequences. Thr 381 has been highlighted as an important residue for substrate selectivity, i.e. shikimic or quinic acid (Singh and Christendat, 2006). We found a Gly instead of Thr 381 in the sequences of groups II and V, and in the FvSDH3 sequence (group I).

DQD/SDHs carry a cofactor-binding motif of three successive amino acids that correspond to Asn 484, Arg 485, and Thr 486 in the AtDQD/SDH sequence. The presence of Arg 485 in this NRT motif attests to NADP(H) binding (Singh *et al.*, 2008). The presence of Asp instead of Arg in the NRT motif is believed to be characteristic of NAD(H)-binding shikimate dehydrogenases (Michel *et al.*, 2003). We noted that only DQD/SDHs from group II exhibit an Asp in the NRT motif.

### *VvSDH* expression pattern, GA and β-G content in grape berry

**Fig. 4.**
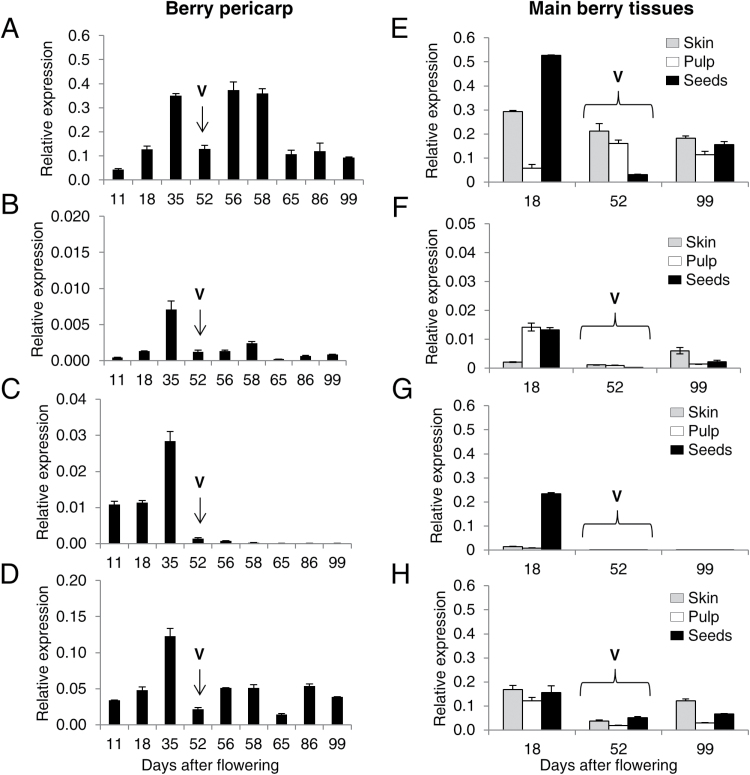
*VvSDH* expression pattern in grape berry. (A–D) qRT-PCR analysis of the expression level of *VvSDH1* (A), *VvSDH2* (B), *VvSDH3* (C), and *VvSDH4* (D) in the pericarp of grape berries harvested at different days after flowering indicated on the *x* axis. Véraison is marked by the arrow. Data represent the mean of three replicates ±SD. (E–H) qRT-PCR analysis of the expression level of *VvSDH1* (E), *VvSDH2* (F), *VvSDH3* (G), and *VvSDH4* (H) in the main tissues of grape berries harvested at different days after flowering indicated on the *x* axis. Green stage, véraison (marked by the brace), and maturity corresponds to 18, 52, and 99 d after flowering, respectively. Grey, white, and black bars correspond to skin, pulp, and seeds, respectively. Data represent the mean of three replicates ±SD.

The expression pattern of each *VvSDH* in the pericarp has been determined during grape berry development via quantitative real time PCR (Fig. 4A). *VvSDH2* and *VvSDH4* expression is relatively stable during grape development, except for a peak of expression before véraison (35 daf). High expression is also observed for *VvSDH1* before véraison (35 daf) but its maximum expression level is reached after véraison (56 daf). Interestingly, the *VvSDH3* expression level rises during the green stage to reach a peak at 35 daf. At véraison, its expression drops and stays very low throughout the ripening stage.

*VvSDH* expression has been also analysed in the main berry tissues (skin, pulp, and seeds) at three development stages (green stage, véraison, and maturity) to determine their spatial and temporal expression patterns (Fig. 4B). Generally, the expression levels of *VvSDH1*, *VvSDH2*, and *VvSDH4* are maximal at the green stage (18 daf), low at véraison, and intermediate at maturity. During the green stage, *VvSDH1* is less expressed in the pulp than in skin and seeds, *VvSDH2* is less expressed in skin than in pulp and seeds, whereas *VvSDH4* expression is relatively high in the three tissues. *VvSDH3* has a very low level of expression whatever the tissue or the developmental stage, except in the immature seeds.

**Fig. 5.**
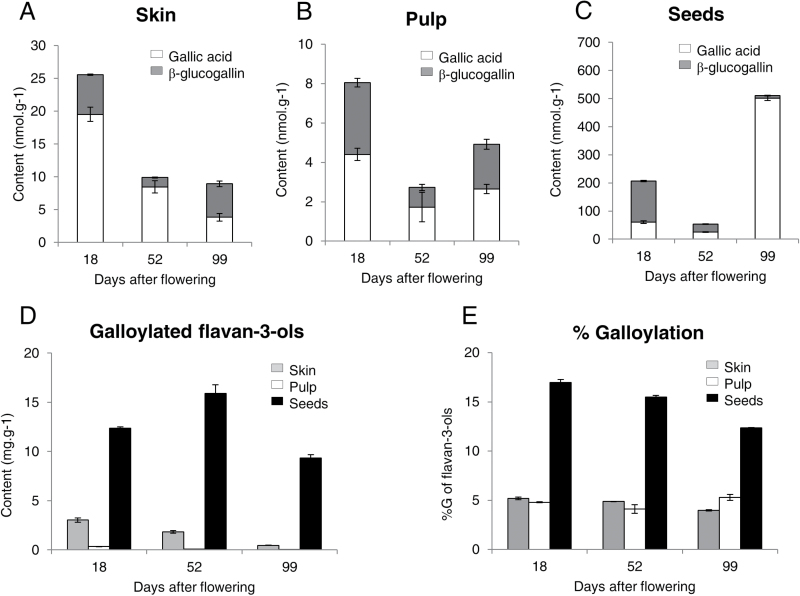
Gallic acid metabolism in grape berry tissues along development. (A–C) Gallic acid and β-glucogallin in grape berry tissues (A), skin (B), pulp, and (C) seeds. White and grey bars correspond to GA and β-glucogallin, respectively. Data are expressed g^–1^ of fresh weight and represent the mean of three replicates ±SD. Samples corresponding to the green stage, véraison, and maturity were collected at 18, 52, and 99 d after flowering, respectively. (D) Galloylated flavan-3-ols in grape berry tissues. Galloylated flavan-3-ols have been quantified after depolymerization by phloroglucinolysis. Grey, white, and black bars correspond to skin, pulp, and seeds, respectively. Data are expressed g^–1^ of frozen powder and represent the mean of three replicates ±SD. (E) Galloylation rate (%G) of flavan-3-ols in grape berry tissues. Flavan-3-ols have been quantified after depolymerization by phloroglucinolysis. Grey, white, and black bars correspond to skin, pulp, and seeds, respectively. Data represent the mean of three replicates ±SD.

The content of galloylated flavan-3-ols and %G, GA and its glucose ester, β-G, was measured in skin, pulp, and seeds, at three development stages: green stage (18 daf), véraison (52 daf), and maturity (99 daf) (Fig. 5). At the same development stage, GA and β-G contents were always higher in seeds than in pulp and skin. The molar ratio β-G/GA was systematically lower or close to 1, except in immature seeds in which it was close to 2.4. In skin and pulp, the GA content was maximal during the green stage (~20 and 4 nmol g^−1^ FW, respectively, Fig. 5A, B). Therefore, GA is mainly synthesized before véraison. The highest content measured in mature seeds (~500 nmol g^−1^ FW, Fig. 5C) could be due to the release of galloyl moieties from galloylated flavan-3-ols, as their content decreases from véraison in seeds (Fig. 5D) leading to a reduction of flavan-3-ols %G (Fig. 5E).

### Enzymatic activity of the recombinant enzymes

GST-tagged VvSDHs have been produced in *E. coli*. After purification and tag removal, the recombinant proteins have been tested *in vitro*. The NADPH-dependent reduction of 3-DHS (reaction 2, Fig. 1) and the NADP^+^-dependent oxidation of SA (reaction 3) have been tested.

The influence of pH on enzymatic assays has been studied. Kinetic parameters (*K*_m_, *V*_max_) were determined at pH 7 to mimic cytosolic conditions and at pH 9, a more alkaline condition closer to the pH of the chloroplast stroma (Song *et al.*, 2004). However, even if the SA pathway is considered to be chloroplastic, NtSDH2 has been localized in the cytosolic compartment in *Nicotiana tabacum* (Ding *et al.*, 2007).

**Fig. 6.**
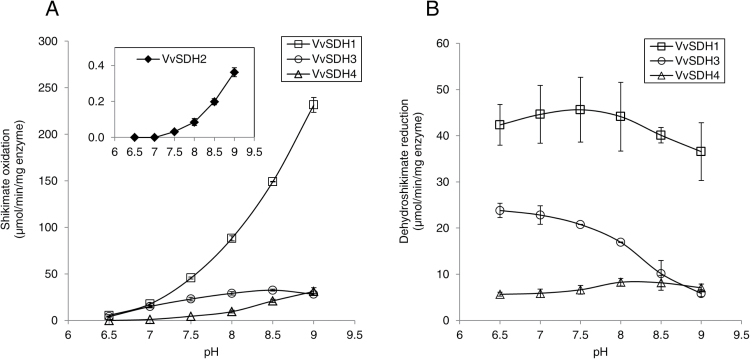
pH effect on VvSDH specific activity. (A) VvSDH activity from shikimic acid and NADP^+^. VvSDH activity was measured by spectrophotometry and recorded NADPH variation 2min after adding the enzyme. Bis-Tris Propane HCl buffer allowed measurements at pH 6.5, 7, 7.5, 8, 8.5, and 9. Data represent the mean of three replicates ±SD. Data are presented independently for VvSDH2 activity due to its lower activity. (B) VvSDH activity from 3-dehydroshikimate and NADPH. VvSDH activity was measured by spectrophotometry and recorded NADPH variation 2min after adding the enzyme. Bis-Tris Propane HCl buffer allowed measurements at pH 6.5, 7, 7.5, 8, 8.5, and 9. Data represent the mean of three replicates ±SD.

For SA oxidation, VvSDH1 and VvSDH4 specific activity increased with pH up to 9, whereas VvSDH3 reached its maximum at pH 8.5 (Fig. 6A). No activity has been detected at pH 7 for VvSDH2 and VvSDH4. For 3-DHS reduction (Fig. 6B), VvSDH3 exhibited a maximal specific activity at pH 6.5 and its activity decreased as the pH increased. VvSDH1 and VvSDH4 reached their maximal specific activity at pH 7.5 and pH 8, respectively.

Substrate and cofactor kinetic parameters have been determined for reactions 2 (3-DHS reduction) and 3 (SA oxidation) at pH 7 and 9, whenever it was possible. Kinetic parameters could not be measured for VvSDH2 due to its low activity.

Considering enzymatic SA oxidation at pH 9, VvSDH3 had higher affinity for SA (*K*_m (SA)_=27.3 µM), compared with VvSDH1 (*K*_m (SA)_=90 µM) and VvSDH4 (*K*_m (SA)_=146.8 µM, Table 1).VvSDH1 had higher affinity for SA at pH 7. *K*_m (SA)_ values are close to those measured for tobacco NtSDH1 (Ding *et al.*, 2007). VvSDH3 and VvSDH4 presented a similar catalytic efficiency (*k*_cat_/*K*_m_) at pH 9 for SA, but this value was about six times lower than VvSDH1 catalytic efficiency under the same conditions. Both VvSDH1 and VvSDH3 had a higher affinity for NADP^+^ at pH 7 than at pH 9.

**Table 1.** Michaelis-Menten kinetic parameters of recombinant VvSDHs

Enzyme	Molecule	pH	*K*_m_ (µM)	*V*_max_ (µkat mg^−1^)	*k*_cat_ (s^−1^)	*k*_cat_/*K*_m_ (mM^−1^ s^−1^)
VvSDH1	NADP^+^	9	214.7±56.4	4.23±0.47	239±26.3	1113
		7	117.2±28.3	0.54±0.05	30.70±3.1	262
	SA	9	90±16	1.67±0.13	94.55±7.2	1051
		7	155±32.6	0.21±0.01	11.72±0.77	76
	3-DHS	7	156.9±31.3	1.70±0.18	95.85±10.19	611
VvSDH2	NADP^+^	9	nd^*a*^			
		7	nd			
	SA	9	nd			
		7	nd			
	3-DHS	7	nd			
VvSDH3	NADP^+^	9	38.4±11.9	0.45±0.05	26.11±2.68	680
		7	23±4.9	0.34±0.02	19.78±1.28	862
	SA	9	27.3±10.4	0.09±0.01	5.16±0.56	189
		7	422.4±88.3	0.13±0.01	7.56±0.65	18
	3-DHS	7	46.3±18.2	0.15±0.02	8.62±0.93	186
VvSDH4	NADP^+^	9	53.8±5.8	0.50±0.02	28.89±1.06	537
		7	nd			
	SA	9	146.8±60	0.46±0.09	26.12±4.97	178
		7	nd			
	3-DHS	7	nd			

^a^ nd, not determined.

At pH 7, VvSDH3 had a higher affinity than VvSDH1 for 3-DHS (*K*_m (3-DHS)_=46.3 µM and 156.9 µM, respectively). However, the catalytic efficiency was about three times higher for VvSDH1 (*k*_cat_/*K*_m_=611) compared with VvSDH3 (*k*_cat_/*K*_m_=186). The VvSDH4 kinetic parameters could not be determined for the NADPH-dependent reduction of 3-DHS due to its low activity at pH 7.

No activity was detected using GST produced by *E. coli* from an empty vector (data not shown).

### GA production by recombinant VvSDH

GA biosynthesis from SA or 3-DHS as the substrate and NADP^+^ as the cofactor (Fig. 1, reaction 4) was measured for each recombinant VvSDH. The identity of the enzymatic products (3-DHS and GA) has been confirmed by an extracted ion chromatogram and fragmentation spectrum compared with the injection of corresponding commercial standards (Supplementary Figs S2, S3).

As the VvSDH specific activity from SA was higher at pH 9, this pH condition was chosen to characterize their capacity to produce GA.

**Fig. 7.**
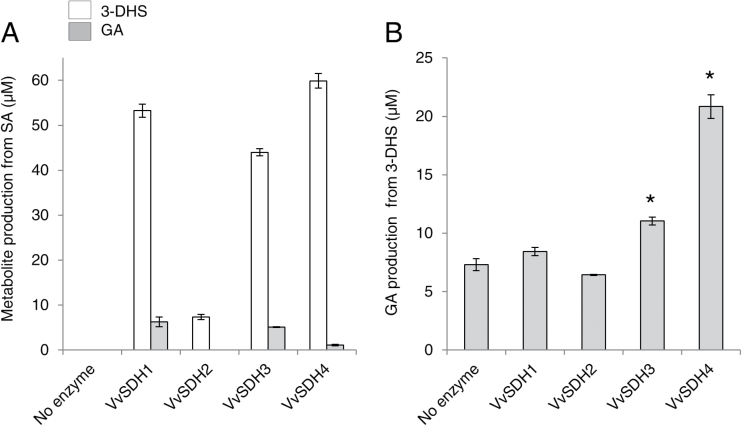
*In vitro* production of 3-dehydroshikimate and gallic acid by recombinant VvSDH. (A) Quantification of 3-DHS (white bars) and GA (grey bars) in conditions lacking the enzyme and in the presence of each VvSDH from shikimic acid and NADP^+^, at pH 9. Data represent the mean of three replicates ±SD. The reaction products in the enzymatic assays were quantified by HPLC using UV-DAD (λ=280nm). (B) GA quantification in conditions lacking the enzyme and in the presence of each VvSDH from 3-DHS and NADP^+^, at pH 9. Data represent the mean of three replicates ±SD. The significance of the results was statistically assessed with a Student’s *t* test using a two-tailed alternative. *: *P* <0.01. The reaction products in the enzymatic assays were quantified by HPLC using UV-DAD (λ=280nm).

After incubation of 4mM SA without enzyme, 3-DHS and GA were not detected in the HPLC chromatogram (Supplementary Fig. S4A). VvSDH2 produced 3-DHS (7.39 µM) but no GA from SA (Fig. 7A). VvSDH1, VvSDH3, and VvSDH4 activity resulted in the formation of both 3-DHS (53.26, 43.99, and 59.89 µM, respectively) and GA (6.26, 5.09, and 1.12 µM, respectively) (Fig. 7A). Concerning GA production, VvSDH1 was the most efficient enzyme from SA. Spontaneous formation of protocatechuic acid (PCA) and GA was detected in the 4mM 3-DHS solution without enzyme (Supplementary Fig. S4B). Their formation increased with pH (data not shown). VvSDH4 and VvSDH3, to a lesser extent, were able significantly to increase GA production compared with the control conditions without enzyme (Fig. 7B). GA production by VvSDH1 and VvSDH2 was not significantly different from the control condition lacking enzyme. *In vitro* assays indicate that GA can be produced from 3-DHS by (i) spontaneous transformation in alkaline conditions and (ii) enzymatic conversion by some of the VvSDHs.

### Metabolic profiling of grapevine hairy-roots over-expressing *VvSDH3*

Aromatic amino acids, hydroxybenzoic and hydroxycinnamic acids, stilbenoids, and flavan-3-ol composition were measured in three independent lines of hairy-roots transformed with *VvSDH3* (numbered 3A, 6A, and 9A) and compared with a control line devoid of transgene (Supplementary Table S4). In spite of different trials, we could not obtain transgenic hairy-roots over-expressing *VvSDH4*, and we suspect a detrimental effect of *VvSDH4* over-expression on root development. The relative expression level of *VvSDH3* has been measured in control and transgenic lines, using *VvEF1α* as the reference gene (Supplementary Fig. S5).

**Fig. 8.**
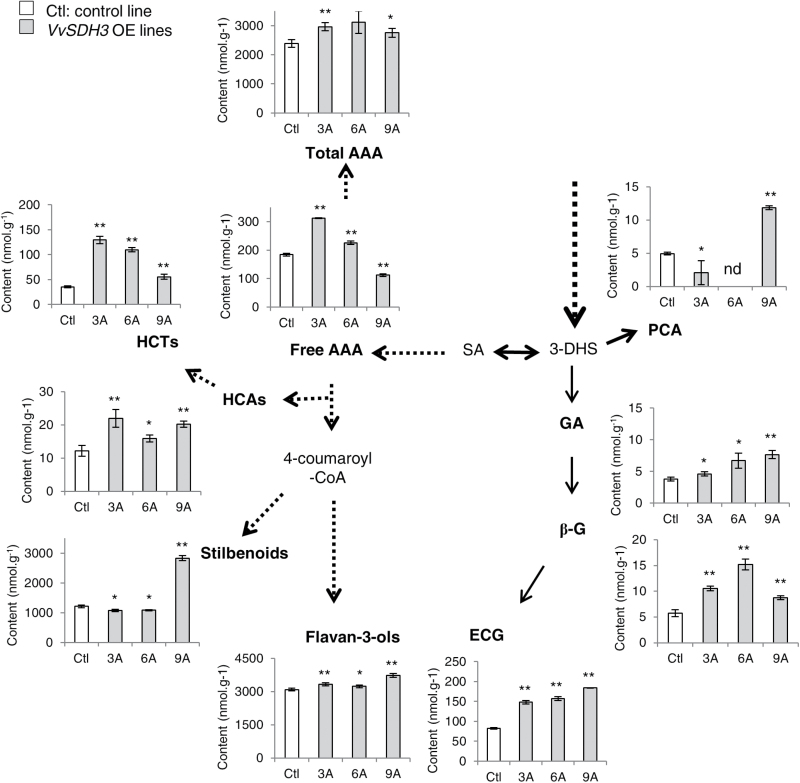
Metabolic profiling of grapevine hairy-roots from VvSDH3-over-expression lines. Control: hairy-root devoid of transgene. Lines 3A, 6A, and 9A are three independent lines transformed with *VvSDH3*. Metabolites are denoted as nmol g^−1^ hairy-root fresh weight. The dotted lines indicate several enzymatic steps. Each data set represents the mean value of three assays ±SD. The significance of the results was statistically assessed with a Student’s *t* test using a two-tailed alternative. *: 0.01<*P* <0.05, **: *P* <0.01. Abbreviations: 3-DHS, 3-dehydroshikimate; SA, shikimic acid; AAA, aromatic amino acids; HCAs, hydroxycinnamic acids; HCTs, hydroxycinnamoyl tartrates; GA, gallic acid; β-G, β-glucogallin; ECG, epicatechin gallate; nd, not determined.

The content of free aromatic amino acids was significantly higher in transgenic lines 3A (+70%) and 6A (+20%), but lower in line 9A (–64%). More precisely, the contents of phenylalanine and tyrosine were significantly higher (except in line 9A) whereas tryptophan was less accumulated in all transgenic lines (Supplementary Table S4). The total content of the aromatic amino acids was significantly higher in line 3A and tended to be higher in lines 6A (+30%) and 9A (+16%) (Fig. 8; Supplementary Table S4).

Hydroxycinnamic acids, in their free form or esterified with tartaric acid (hydroxycinnamoyl tartrates), were significantly more accumulated in all transgenic lines compared with the control line (Fig. 8; Supplementary Table S4).

Compared with the control line, the PCA content tended to be lower in 3A and could not be detected in line 6A (Fig. 8). By contrast, line 9A contained a significantly higher PCA pool.

Lines 3A and 6A tended to accumulate more GA than the control line (+22% and +77%, respectively) and 9A contained significantly more GA than the control line. All transformed lines contained significantly more β-G (up to +165% for line 9A) and galloylated flavan-3-ols (between +80% and +124%) than the control line. %G was significantly higher in the three lines over-expressing *VvSDH3* compared with the control line.

The total content of flavan-3-ols was significantly higher in lines 3A and 9A transformed with *VvSDH3*, but was equivalent in line 6A (Fig. 8). The contents of stilbenoids were lower in transgenic lines 3A and 6A (about –10%) but significantly higher in line 9A.

## Discussion

Among the four VvSDHs, VvSDH1, VvSDH3, and VvSDH4 proteins exhibited a ‘classical’ SDH activity from SA, using NADP^+^ as the cofactor. By contrast, VvSDH2 exhibited a very low ‘classical’ SDH activity compared with the other VvSDHs. VvSDH2 clustered in group II with NtSDH2 and the characterized poplar DQD/SDHs that did not exhibit ‘classical’ SDH activity, but rather quinate dehydrogenase activity from quinate and NAD^+^ (Poptr2 and -3, Guo *et al.*, 2014). Ding *et al.* (2007) have suggested that ‘the *in vivo* preferred substrate of NtSDH2 is in fact a derivative of shikimate exhibiting a larger functional group at the C1 position ring’, that corresponds to the description of quinate. Interestingly, this protein has been localized in the cytosolic compartment. Due to the absence of SDH activity and high sequence identity with Poptr2 and Poptr3, VvSDH2 is a good candidate for the NAD^+^-dependent biosynthesis of quinic acid. The *In vitro* activity of VvSDH2 must be further examined to check if this enzyme exhibits quinate dehydrogenase activity.

Specific activity for SA oxidation reached a maximum at a pH close to 9. Similar measurements have been reported with plant DQD/SDHs (Ding *et al.*, 2007; Singh and Christendat, 2007). We observed that the pH optimum was more acidic for 3-DHS reduction. *K*_m (SA)_ and *K*_m (NADP+)_ values at pH 9 were close to those measured for NtSDH1 (Ding *et al.*, 2007). At pH 7, *K*_m (3-DHS)_ is close to *K*_m (SA)_ for VvSHD1, indicating that this enzyme has similar affinities for its substrate in both reaction directions. We did not find any studies reporting *K*_m (3-DHS)_ from heterologously produced plant DQD/SDHs, *K*_m_ being determined from SA only. However, the SDH activity of DQD/SDHs has been observed with purified enzymes from pea (Mousdale *et al.*, 1987) and a *K*_m (3-DHS)_=210 µM (with 0.35mM NADPH, at pH 7) was reported, close to the values obtained in the present work with VvSDHs.

VvSDH1 exhibited the highest ‘classical’ SDH activity. Concerning GA biosynthesis, VvSDH1 produced the highest amount of GA from SA but GA production with 3-DHS as substrate was not significantly different from the condition without enzyme. Thus, the assayed GA with SA as the substrate could result from the spontaneous conversion of the enzymatically formed 3-DHS, rather than from enzymatic activity. During grape berry development, *VvSDH1* was highly expressed both before and after véraison, in contrast to the other *VvSDHs*. Even if this gene was mainly expressed in skin and seeds during the green stage, its relative expression was similar in the three tissues at véraison and maturity. We can hypothesize that, *in planta*, VvSDH1 channels carbon flux towards aromatic amino acids biosynthesis in the main trunk of the SA pathway, efficiently converting 3-DHS to SA whatever the pH, and SA to 3-DHS more efficiently at higher pH, fitting with chloroplastic conditions. The proteins clustering in group I are essential for optimal plant metabolism and development. Indeed, the silencing of NtSDH1 triggered the retardation of plant growth and a reduction in the aromatic amino acids (Ding *et al.*, 2007). This idea is also supported by the fact that all the species examined, except persimmon and tea, carry a DQD/SDH from group I. However, it is likely that these two species, whose complete genome sequencing is not yet available, carry other DQD/SDH genes.

VvSDH3 and VvSDH4 exhibited lower ‘classical’ SDH activity from SA and NADP^+^ than VvSDH1 and Poptr1 (Guo *et al.*, 2014). However, *in vitro* assays showed that these enzymes catalyse GA production. Muir *et al.* (2011) reported that JrSDH produced similar amounts of GA in similar *in vitro* reaction conditions but after a much longer reaction time (16h instead of the 2h here). From the same metabolite (3-DHS), GA production was lower than SA production. This feature is consistent with the fact that enzymes involved in specialized metabolite biosynthesis are less efficient than those involved in primary metabolism (Milo and Last, 2012). Non-enzymatic formation of PCA from 3-DHS has been reported earlier (Richman *et al.*, 1996; Li *et al.*, 1999) and has been observed in alkaline conditions (pH 10: Ossipov *et al.*, 2003).

Interestingly, VvSDH4 clustered with a DQD/SDH identified in persimmon (DkSDH). In non-astringent persimmon fruits containing low levels of galloylated PAs, this DkSDH is down-regulated compared with astringent persimmon fruits (Akagi *et al.*, 2009). The species possessing DQD/SDH clustered in groups III or IV are known to produce high amounts of hydrolysable tannins (strawberry, eucalyptus) or galloylated flavan-3-ols (tea plant, grape, persimmon). By contrast, the species devoid of DQD/SDHs from groups III and IV, such as Arabidopsis, tobacco, tomato, and orange, do not produce galloylated tannins.

It has been postulated that the C-terminal SDH domain of the bifunctional enzyme DQD/SDH in plants could be involved in GA biosynthesis (Muir *et al.*, 2011). Substrate binding and orientation can be influenced by the substitution of several key amino acids. It could explain why VvSDH3 and VvSDH4, exhibiting some divergences with AtSDH for some of those important amino acids, acquired the capacity to produce GA. However, structure–function relationships need to be experimentally demonstrated, notably by site-directed mutagenesis.

The relative expression patterns of *VvSDH3* and *VvSDH4* (higher before véraison) was synchronous with GA biosynthesis in grape berry. Otherwise, the highest expression of these genes was detected in tissues in parallel with the biosynthesis of galloylated PAs (Kennedy *et al.*, 2000; Bogs *et al.*, 2005): immature seeds for *VvSDH3*, immature skin and seeds for *VvSDH4*. VvSDH3 and VvSDH4 probably have a minor role in the SA pathway compared with VvSDH1, due to their lower ‘classical’ SDH activity, but possibly orient a small part of the carbon flux towards GA metabolism. Actually, *VvSDH3* over-expression in grapevine hairy-roots resulted in an increase in GA, β-G, and flavan-3-ols %G. A part of the generated GA pool is glucosylated and transferred to flavan-3-ols, leading to an accumulation of GA as galloylated flavan-3-ols. PCA, another hydroxybenzoic acid directly formed from 3-DHS (Dewick, 2002) is less accumulated in transgenic hairy-roots (except in line 9A, Supplementary Table S4).

*VvSDH3* over-expression did not affect carbon flux into the SA pathway. Indeed, the content of GA-derived metabolites is low (in the order of nmol g^−1^) compared with the end products derived from the SA pathway such as flavan-3-ols and stilbenoids (Supplementary Table S4) which is in the order of µmol g^−1^. By contrast, the ‘classical’ SDH activity of VvSDH3 could even be responsible for the increased accumulation of aromatic amino acids, generated by the last steps of the SA pathway, and hydroxycinnamic acids derivatives in the over-expressing lines. Downstream biosynthesis pathways are less impacted since the contents of stilbenoids and flavan-3-ols are equivalent in transgenic and control hairy-roots.

*In planta*, VvSDH3 activity towards the classical SA pathway or GA production is influenced by the redox state in the cellular compartment where it is located and, more precisely, the NADP^+^/NADPH ratio. In the presence of NADPH, *VvSDH3* efficiently converts 3-DHS into SA. In the presence of NADP^+^, *VvSDH3* converts 3-DHS into GA, generating a leak in the SA pathway.

The lack of a plastidic target peptide for some VvSDH questions the intracellular localization of these enzymes. Future experiments with grapevine hairy-roots, transformed with VvSDH fused to GFP, could be used to localize them at the cellular level. VvSDH3 and VvSDH4 are good candidates for GA biosynthesis in immature grape berry tissues. However, other molecular actors involved in GA transport and PA galloylation remain to be identified.

To conclude, we have shown that the SA pathway and GA metabolism in grapevine are tightly related to the activity of VvSDHs.

## Supplementary data

Supplementary data can be found at JXB online.

**Figure S1**. Genomic structure of *VvSDHs*.

**Figure S2**. UPLC-DAD-MS analysis of 3-dehydroshikimate in enzymatic assays.

Figure S3. UPLC-DAD-MS analysis of gallic acid in enzymatic assays.

Figure S4. HPLC chromatograms (λ=280nm) of the reaction assay.

Figure S5. *VvSDH3* relative expression level in hairy-roots.

Table S1. Primers used for *SDH* cloning and quantitative real time PCR.

Table S2. Sequence identity of grapevine shikimate dehydrogenases (VvSDHs).

Table S3. Summary of plant species used to analyse DQD/SDH sequences.

Table S4. Metabolic profiling of grapevine hairy-roots.

Alignment S1. Multiple sequence alignment of DQD/SDH proteins.

Supplementary Data
