# Supplementary material for: Two shikimate dehydrogenases, VvSDH3 and VvSDH4, are involved in gallic acid biosynthesis in grapevine
Source: J Exp Bot. 2016 May 28;67(11):3537–50. doi: 10.1093/jxb/erw184 (PMC4892741; doi:10.1093/jxb/erw184)
Supplement: Supplementary Data [file supp_67_11_3537__index.html]

Two shikimate dehydrogenases, VvSDH3 and VvSDH4, are involved in gallic acid biosynthesis in grapevine — Supplementary Data 

# Two shikimate dehydrogenases, *VvSDH3* and *VvSDH4*, are involved in gallic acid biosynthesis in grapevine

## Supplementary Data

Data files

- Supplementary\_figures\_S1\_S5\_tables\_S1\_S5\_alignmentS1.pdf - Supplementary Data
