# Supplementary material for: Two shikimate dehydrogenases, VvSDH3 and VvSDH4, are involved in gallic acid biosynthesis in grapevine
Source: J Exp Bot. 2016 May 28;67(11):3537–50. doi: 10.1093/jxb/erw184 (PMC4892741; doi:10.1093/jxb/erw184)
Supplement: Supplementary Data [file supp_erw184_Supplementary_figures_S1_S5_tables_S1_S5_alignmentS1.pdf]

Two shikimate dehydrogenases, *VvSDH3* and *VvSDH4*, are involved in gallic acid biosynthesis in grapevine

Thibaut Bontpart, Thérèse Marlin, Sandrine Vialet, Jean-Luc Guiraud, Lucie Pinasseau, Emmanuelle Meudec, Nicolas Sommerer, Véronique Cheynier, Nancy Terrier

SUPPLEMENTARY DATA

Figure S1. Genomic structure of *VvSDHs*.

The exon-intron pattern was recovered from the 12X version of *Vitis vinifera* genome with gene prediction V2 (http://genomes.cribi.unipd.it/grape/). The scale indicates the chromosome and the genomic region shown.

Light grey and dark grey boxes indicate the putative UTR regions and exonic sequences, respectively.

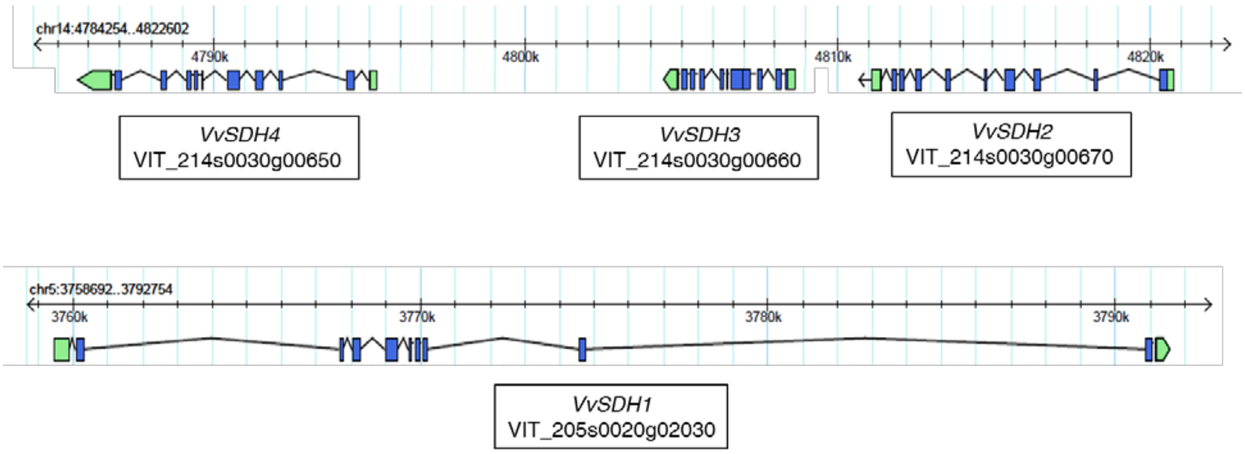

Figure S2. UPLC-DAD-MS analysis of 3-dehydroshikimate in enzymatic assays.

Extracted-ion chromatogram at m/z 171 in the negative ion mode in enzymatic assay without enzyme from (A) shikimic acid and NADP$^+$, (B) 3-dehydroshikimate and NADP$^+$; and in presence of VvSDH3 from (C) shikimic acid and NADP$^+$, (D) 3-dehydroshikimate and NADP$^+$. The corresponding mass spectra and fragmentation by MS2 of 171 ion yielding a fragment ion at m/z 127 is indicated by the same letter with an apostrophe.

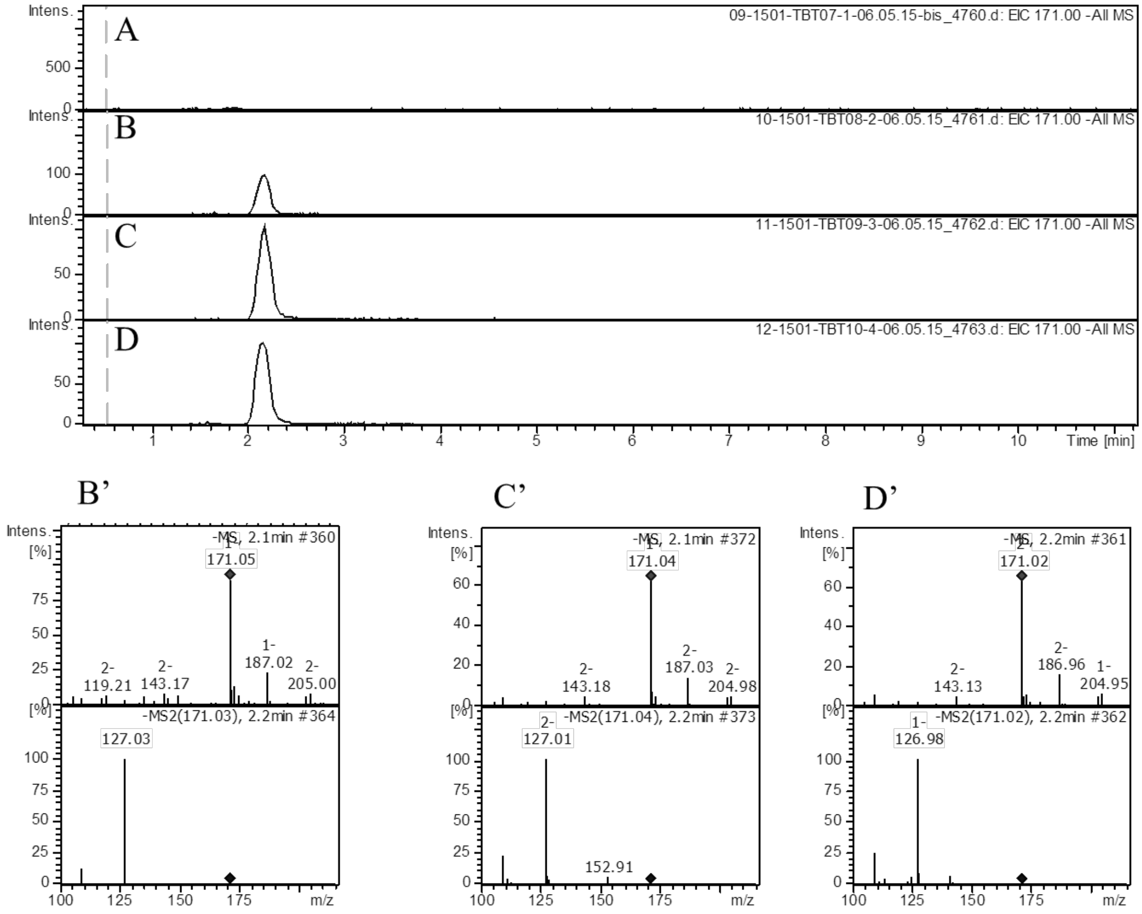

Figure S3. UPLC-DAD-MS analysis of gallic acid in enzymatic assays. Extracted-ion chromatogram at m/z 169 in the negative ion mode in enzymatic assay without enzyme from (**A**) shikimic acid and NADP$^+$, (**B**) 3-dehydroshikimate and NADP$^+$; and in presence of VvSDH3 from (**C**) shikimic acid and NADP$^+$, (**D**) 3-dehydroshikimate and NADP$^+$. The corresponding mass spectra and fragmentation by MS2 of 169 ion yielding a fragment ion at m/z 125 is indicated by the same letter with an apostrophe.

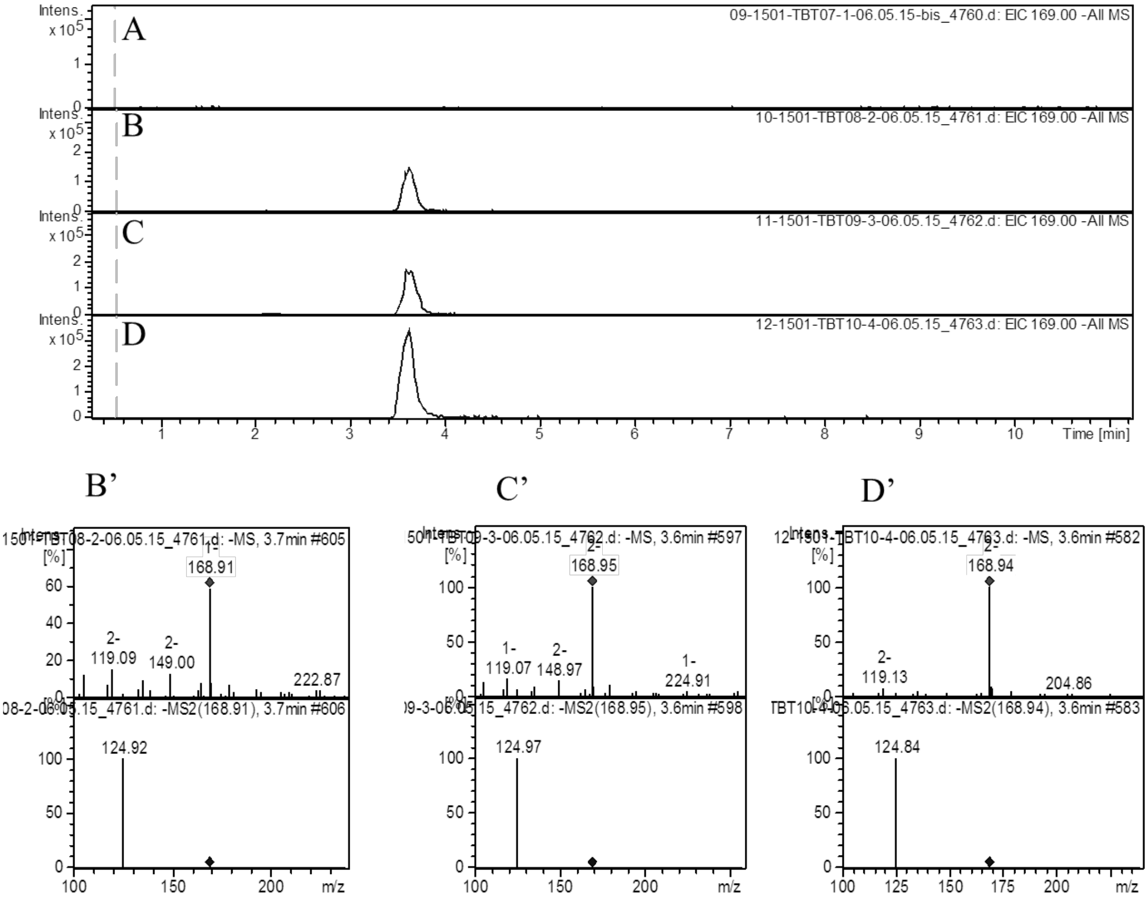

Figure S4. HPLC chromatograms (λ = 280 nm) of reaction assay.

(**A**) Control condition (blue line) contained Bis-Tris Propane pH 9 100 mM, 4 mMSA, 2 mM NADP$^+$, 8 mM ascorbic acid. Enzymatic assay (red line) contains the same mixture and 1 µg VvSDH1 were added to start the reaction.

(**B)** Control condition (blue line) contained Bis-Tris Propane pH 9 100 mM, 4 mM3-DHS, 2 mM NADP$^+$, 8 mM ascorbic acid. Enzymatic assay (red line) contains the same mixture and 1 µg VvSDH4 were added to start the reaction.

Peak number: **1**: Coelution of ascorbic and shikimic acids, **2**: 3-dehydroshikimate, **3**: NADP$^+$, **4**: Gallic acid, **5**: Protocatechuic acid.

A

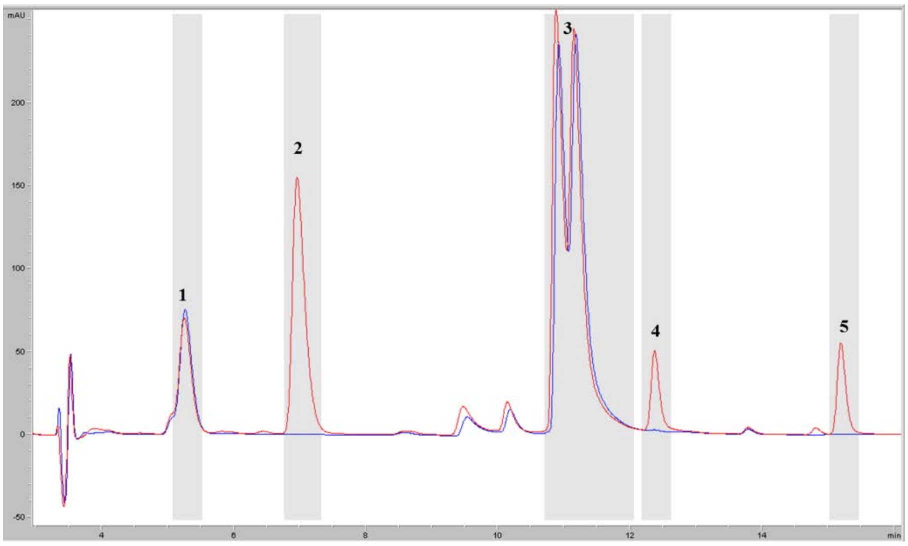

B

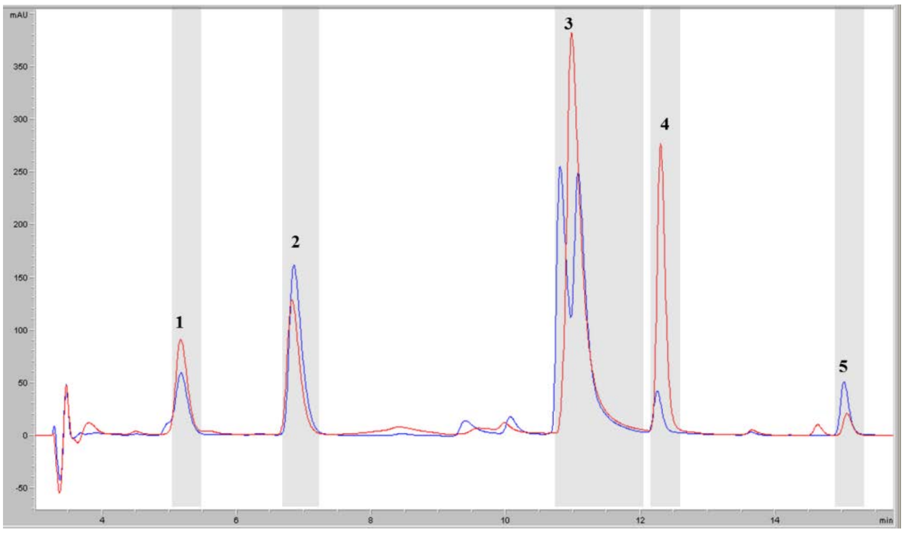

Figure S5. *VvSDH3* relative expression level in hairy-roots.

VvSDH3 expression was determined by real-time PCR and normalized with the expression of the reference gene *Elongation factor 1 α* (*EF1α*). Control line is devoid of transgene. The lines 3A, 6A and 9A are three independent transgenic lines transformed with the construct pH2GW7-VvSDH3. Data represent the mean of three replicates ± SD.

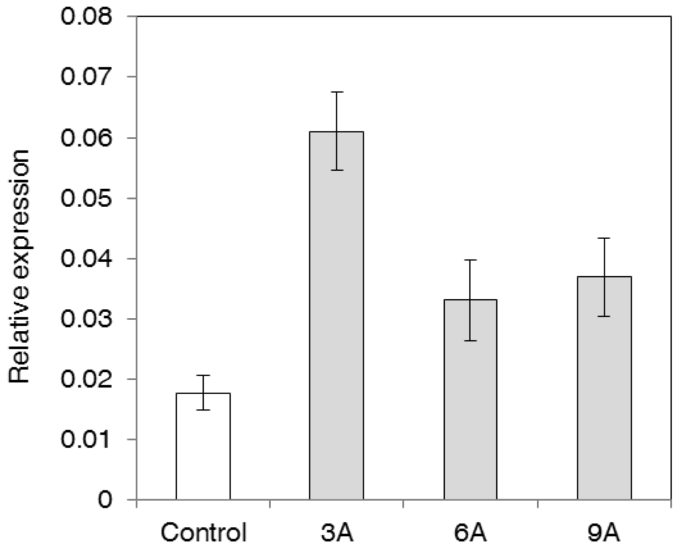

Table S1. Primers used for *SDH* cloning and quantitative polymerase chain reaction (qPCR).

Purpose	Name	5'-3' sequence	Sense	Restriction site
pGEMT	SDH1pGEXstart	GGATCC**ATG**GAAAGCGGAGGAATGAG	F	BamHI
Easy cloning	SDH1pGEXstop	CTCGAGTTATTGCAAATTTGATATGAATTGCTT	R	XhoI
	SDH2pGEXstart	GGATCC**ATG**GATGATGTTGGAGTTTTGAA	F	BamHI
	SDH2pGEXstop	CTCGAGTCAGAACTTTGATAAAATAATCTCC	R	XhoI
	SDH3pGEXstart	GGATCC**ATG**GGGAGCCTCCCATTTACTGT	F	BamHI
	SDH3pGEXstop	CTCGAGTTATGCGTGTTTCGACATAAGCTC	R	XhoI
	SDH4pGEXstart	GGATCCGGAGCCCGGAGAAATTCG	F	BamHI
	SDH4pGEXstop	CTCGAGTTATGTATTCCTCACCAAAACTTCC	R	XhoI
pH2GW7	SDH3TopoStart	CACC**ATG**GGGAGCCTCCCATTTACTGT	F	
cloning	SDH3TopoStop	TTATGCGTGTTTCGACATAAGCTCC	R	
	SDH4TopoStart	CACC**ATG**ACTCTCAGCAGCGTTCCG	F	
	SDH4TopoStop	TTATGTATTCCTCACCAAAACTTCC	R	
Quantitative	SDH1Q	GGCAGGCATATGAGCAGTTT	F	
PCR grape	SDH1Q	CAGCTCTCAACAGGACAGCTC	R	
berry	SDH2Q	AGTTCTGATACACTGCCTCATG	F	
	SDH2Q	ACCCTTATTGAAATGTGCTTGGA	R	
	SDH3Q	AGAGATGTTGATCCGCCAAG	F	
	SDH3Q	AACTGACTGCCCAAGCAAAT	R	
	SDH4Q	TCAGGGAAGTTTTGGTGAGGA	F	
	SDH4Q	ACTCAGCAGCTAATCACAAAGA	R	
Quantitative	SDH3OEQ	AGTTGCCAACCGAACATTTGA	F	
PCR hairy-roots	SDH3OEQ	GCTTGGGAATGGGAGTGTCA	R	

Start codon is in bold letters. Restriction site is underlined.

Table S2. Sequence identity of grape shikimate dehydrogenases (VvSDHs).

Amino acids and nucleotides (between brackets) identity is expressed in percentage and has been defined using Clustal Omega with default parameters (http://www.ebi.ac.uk/Tools/msa/clustalo/) from cDNA sequences cloned from Syrah cultivar.

	**VvSDH1**	**VvSDH2**	**VvSDH3**	**VvSDH4**
**VvSDH1**	100	51.45 (59.17)	69.75 (72.44)	70.33 (74.68)
**VvSDH2**		100	46.82 (58.14)	48.94 (57.95)
**VvSDH3**			100	70.62 (74.62)
**VvSDH4**				100

Table S3. Summary of plant species used to analyze DQD/SDH sequences.

Species	Common name	Family	ID	Accession n°
Arabidopsis thaliana	Arabidopsis	Brassicaceae	AtSDH	AAF08579
Camellia sinensis	Tea plant	Theaceae	CasSDH1	AIZ93902
			CasSDH2	AJA40947
			CasSDH3	AJA40948
Diospyros kaki	Persimmon	Ebenaceae	DkSDH1	BAI40147
Eucalyptus grandis	Eucalyptus	Myrtaceae	EgSDH1	Eucgr.H01214.1
			EgSDH2	Eucgr.H04428.1
			EgSDH3	Eucgr.H04427.1
			EgSDH4	Eucgr.B01770.2
			EgSDH5	Eucgr.J00263.6
Fragaria vesca	Strawberry	Rosaceae	FvSDH1	XP_004302480
			FvSDH2	XP_004302479
			FvSDH3	XP_004289250
			FvSDH4	XP_004288087
Juglans regia	Walnut	Juglandaceae	JrSDH	AAW65140
Nicotiana tabacum	Tobacco	Solanaceae	NtSDH1	AAS90325
			NtSDH2	AAS90324
Populus trichocarpa	Poplar	Salicaceae	Poptr1	Potri.010G019000
			Poptr2	Potri.013G029900
			Poptr3	Potri.005G043400
			Poptr4	Potri.014G135500
			Poptr5	Potri.013G029800
Solanum lycopersicum	Tomato	Solanaceae	SlSDH1	AAC17991
			SlSDH2	XP_010327280
			SlSDH3	XP_004242317
Citrus sinensis	Orange	Rutaceae	CsSDH1	orange1.1g010050m
			CsSDH2	orange1.1g010101m
			CsSDH3	orange1.1g007151m
Vitis vinifera	Grapevine	Vitaceae	VvSDH1	KU163040
			VvSDH2	KU163041
			VvSDH3	KU163042
			VvSDH4	KU163043

Table S4. Metabolic profiling of grapevine hairy-roots.

	**Control**	**3A**	**6A**	**9A**
**Free aromatic amino acids**				
Phenylalanine	87.5 ± 1.3	171.0 ± 1.75 **	125.3 ± 1.88 **	93.2 ± 2.11 *
Tyrosine	23.9 ± 0.5	93.1 ± 2.0 **	61.8 ± 2.9 **	nd
Tryptophane	72.7 ± 5.8	47.9 ± 1.3 *	39.0 ± 2.9 **	19.0 ± 5.4 **
**Hydroxybenzoic acids and derivatives**				
Protocatechuic acid	4.97 ± 0.21	3.11 ± 0.25 *	nd	11.88 ± 0.29 **
Gallic acid	3.78 ± 0.31	4.60 ± 0.33 *	6.70 ± 1.20 *	7.65 ± 0.65 **
β-glucogallin	5.74 ± 0.66	10.57 ± 0.47 **	15.18 ± 1.05 **	8.77 ± 0.38 **
**Hydroxycinnamic acids and derivatives**				
Caffeic acid	11.68 ± 1.07	20.92 ± 1.57 **	14.68 ± 1.0 *	19.80 ± 0.5 **
Ferulic acid	nd	0.58 ± 0.54	nd	nd
p-coumaric acid	0.84 ± 0.26	0.77 ± 0.50	1.12 ± 0.06	0.70 ± 0.02
Caftaric acid	14.31 ± 0.27	26.33 ± 1.04 **	25.33 ± 0.86 **	13.76 ± 0.65
Fertaric acid	3.07 ± 0.34	12.92 ± 0.54 **	10.40 ± 1.61 *	5.14 ± 0.16 **
Coutaric acid	17.84 ± 1.42	90.10 ± 5.73 **	73.97 ± 2.03 **	36.43 ± 4.27 *
**Stilbenoids**				
Trans-resveratrol	295.8 ± 17.7	211.8 ± 2.1 *	229.7 ± 3.3 *	618.6 ± 14.6 **
Cis-resveratrol	8.28 ± 0.50	7.99 ± 0.10	5.27 ± 0.09 **	22.87 ± 1.61 **
ε-viniferin	15.00 ± 0.82	18.35 ± 0.66 **	11.65 ± 2.83	105.98 ± 3.24 **
Trans-piceid	371.8 ± 6.9	503.7 ± 16.5 **	458.1 ± 4.3 **	1231.8 ± 52.5 **
Cis-piceid	501.1 ± 18.7	308.1 ± 14.8 **	365.1 ± 6.1 **	838.3 ± 10.1 **
Piceatannol	5.01 ± 0.28	4.15 ± 0.25 *	3.20 ± 0.67 *	0.74 ± 0.1 **
Piceatannol-glucoside	17.89 ± 1.27	19.23 ± 1.84	14.16 ± 2.10	14.27 ± 0.26 *
**Flavonoids**				
Galloylated flavan-3-ols	0.082 ± 0.002	0.148 ± 0.004 **	0.157 ± 0.005 **	0.184 ± 0.001 **
%G flavan-3-ols	2.65 ± 0.01	4.45 ± 0.05 **	4.84 ± 0.07 **	4.93 ± 0.09 **

Control: hairy-root devoid of transgene. Lines 3A, 6A and 9A are three independent lines transformed with *VvSDH3*. Metabolites contents are reported as nmol.g^{-1} hairy-root fresh weight.

Galloylated flavan-3-ols content was calculated as the sum of epicatechin gallate and epicatechin gallate with phloroglucinol adduct. % galloylation (%G) of flavan-3-ols was calculated as a molar ratio.

Each data represents the mean value of 3 assays ± SD. The significance of the results was statistically assessed with a Student's t-test using two-sided alternative. *: $0.01<P<0.05$, **: $P<0.01$.

Alignment S1. Multiple sequence alignment of DQD/SDH proteins. Sequences from grapevine were aligned with sequences from selected dicot species. Alignments were performed with ClustalW.

```
AtSDH        MAASSTNARLTNPPRLLSKPRLSPTSVANLRFPAADFSTRFFADSSSPRLRSVPFPVVFS
NtSDH1       ------------------------------------------------------------
SlSDH1       ------------------------------------------------------------
FvSDH3       ------------------------------------------------------------
Poptr1       ------------------------------------------------------------
FvSDH4       ------------------------------------------------------------
JrSDH        ------------------------------------------------------------
VvSDH1       ------------------------------------------------------------
EgSDH5       ------------------------------------------------------------
CsSDH3       ------------------------------------------------------------
CasSDH2      ------------------------------------------------------------
VvSDH3       ------------------------------------------------------------
EgSDH3       ------------------------------------------------------------
FvSDH1       ------------------------------------------------------------
FvSDH2       ------------------------------------------------------------
Poptr5       ------------------------------------------------------------
CasSDH3      ------------------------------------------------------------
DkSDH        ------------------------------------------------------------
EgSDH2       ------------------------------------------------------------
VvSDH4       ------------------------------------------------------------
CasSDH1      ------------------------------------------------------------
NtSDH2       ------------------------------------------------------------
SlSDH2       ------------------------------------------------------------
EgSDH4       ------------------------------------------------------------
Poptr2       ------------------------------------------------------------
Poptr3       ------------------------------------------------------------
VvSDH2       ------------------------------------------------------------
CsSDH1       ------------------------------------------------------------
SlSDH3       -----------------------------MFPPL-YKRERWSVKEERDII----------
EgSDH1       ------------MAKLHATPQASRAGESKREYPI-HRERSWTVSPPDHWRKQVA--EGVT
Poptr4       ------------------------------------------------------------
CsSDH2       ------------------------------------------------------------


AtSDH        DQRRRRSMEPSNVYVASNSTEMEIGSHDIVKNPSLICAPVMADSIDKMVIETSKAHELGA
NtSDH1       ------------MELVVDSGVRKMEGEAMTRNETLICAPIMADTVDQMLNLMQKAKISGA
SlSDH1       ------------MELVVDSGVKKMEGEAMRKNQTLICAPIMADSVDQMLILMQKAKISGA
FvSDH3       ----------------------------MMGSSTLVCAPIMAETVHKMVRDMSKARDLGA
Poptr1       ---MDSASNVLLASSPSAAAAVGMGSGGVRRNPTLICTPIMADSVDKMAILMAEAKSVGA
FvSDH4       -----------MDSPTLTVASAQVGGGGMRKSSTLICAPIMAESVAKMVVEMGRAKAVGA
```

```
JrSDH        ---------------------------------TLVCAPIMAESVDKMVINMNKAKQGDA
VvSDH1       -----------------------MESGGMSKNSTLICVPIMGETIEKMVVDMSKAKTSGA
EgSDH5       -----------------------MESGAVRKNSTLICVPVMADSVEEMVIQLDKAKSSGA
CsSDH3       ----------MESPNLLVASGSKLVSGGMRKNPTLICVPIMGESVDKMVVDMGKANASGA
CasSDH2      ---------MASGSFSFATSDVQTSSSGVRSGPTLLCTPLIGTTVDQMLTDMRKAKEIGA
VvSDH3       -----------MGSLPFTVSDLQTSVSGVRSNPTLLCTPLMGTTVEQMLTEMRKAKEIGA
EgSDH3       -----------MGSVPFTTSDLQTSTSGFRSSPTLLCTPLMGTTVDQMLIEMRKAKEIGA
FvSDH1       -----------MGSLPFTTSDLHTSTGGFLSSPTLLCTPLMGTTVDQMLIEMHKAKEIGS
FvSDH2       ---------MTLSSIPLVASDLQISY-GTGRNSTLICAPVMGESVDQMLRQLQQAKELGA
Poptr5       -------------------MDLQSAD-GERRNSTLICAPIMAESVDQMLVQMKRAKELGA
CasSDH3      ----------------------------------MICAPVMAENVDQMLLMMRKAKELGA
DkSDH        ----------------------------------MICAPVMAETAEQMLGQMRKAKELGA
EgSDH2       ---------MTLSSIPLTAADLQIPA-GGRRNSTLLCAPVMGESVDQMLGQIRAAKEQGA
VvSDH4       ---------MTLSSVPLATSDIQIPE-GARRNSTLICVPIMADSVDQMLGQIRKAKEVGG
CasSDH1      -----------------------MGSVGVLTNSTTICAPLMSQSVEQMVSDMNQAKAQGA
NtSDH2       -----------------------MGSVGLLKNSAMVCAPLMAPSVEQLVHGMLQAKAQGA
SlSDH2       -----------------------MGSVGLLKNSAMVCAPLMATSVDQLIDEMVEAKSQGA
EgSDH4       ----------------------MGSLSLSSVGLTMVCAPVMGESVDQVVEEMHKAKAQGA
Poptr2       -----------------------MGRAGILANSTMVCAPLMARSVEQMVIDMQSAEAQGA
Poptr3       -----------------------MGSVGVLTNSVMVCAPLMAQSVEQMVIDMHSAKAQGA
VvSDH2       -----------------------MDDVGVLKKETMICTPLMGQSVEQMVRDMHKAKVEGA
CsSDH1       -----------------------MGVVNITKNTTMICAPLMAQSVEQVLSNMYQAKAEGA
SlSDH3       ---------------------------MGLKNDLVVYTRLECESCEEMTCCIEKAKEEGA
EgSDH1       AQTLHSSSR---IFFLHCPSSRRERPREMDRNGVLVCAPLECETLEGMLSSMDKAKAHGA
Poptr4       ---------------------------MAFKNNLLVCTPLECETAGEMLSSMKRAETEGA
CsSDH2       -------------------------MEVAAKNSLLVCTQLECETTEEMQASIEQAKVEGA


AtSDH        DLVEIRLDWLKDFNPLEDLKTIIKKSPLPTLFTYR-------------------PKWEGG
NtSDH1       DLVEVRLDSLKSFNPQSDIDTIIKQSPLPTLFTYR-------------------PTWEGG
SlSDH1       DLVEVRVDSLKSFNPRPDIDTLIKQCPLPTLFTYSYVLGVGQGILLIRYYKGIGPTWEGG
FvSDH3       DVVEIRLDYLKVFNSNQDLKTLIKESPMPTLFTYR-------------------PKWEGG
Poptr1       DLVEIRLDSLKDFNPNSDIKTLILHSPLPTLFTYR-------------------PMWEGG
FvSDH4       DLVEIRLDHLKVFDSSEDVKTLIDQSPLPTLFTYR-------------------PKWEGG
JrSDH        DLVEIRLDSLKSFNPSNDLKTIIKASPLPTLFTYR-------------------PKWEGG
VvSDH1       DLVEVRLDTLKRFNPRQDLEVLIRKCPLPTLFTYR-------------------PKWEGG
EgSDH5       DLVEIRVDGLKNLSPHEDLKTLIKASALPTLFTYR-------------------PKWEGG
CsSDH3       DLVEIRLDGLKNFNPRENIKTLIKESPVPTLFTYR-------------------PIWEGG
CasSDH2      DMVEIRLDCLREFNPRPDLQILIKQSPLPTLVTYR-------------------PIWESG
VvSDH3       DIVEIRLDCLRNFSPAQDLQILIKQSPLPTLVTYR-------------------PIWEGG
EgSDH3       DLVEIRLDCLRNFNPHQDLQILIKQSPLPTLVTYR-------------------PVWEGG
FvSDH1       DVVEIRLDCLRNFNPSSDLQILIKQSPLPTLVTYR-------------------PVWEGG
FvSDH2       DLVEIRLDYIKNFSPRQDLETLIKRSPLPTLVTYR-------------------PKWEGG
Poptr5       DVAEVRVDFLKNFSPRNDLEALIKQCPLPTLITYR-------------------PKWEGG
CasSDH3      DLVEVRIDYLKNFSPHHDLQILIKQCPLPTLITYR-------------------PTWEGG
DkSDH        DLVEIRIDYLKSFSPQQHLEVLIKQSPLPTIITYR-------------------PTWEGG
```

```
EgSDH2      DLVEVRLDFLKSFSPKQDLEILLKQSALPTLVTYR-------------------PKWEGG
VvSDH4      DLVEIRLDYLKNFSPRQDLQFLVKQSPLPTLVTYR-------------------PTWEGG
CasSDH1     DVVEIRLDCIKDFQPNRDLQILLNNKPLPVLIVYR-------------------PQWEGG
NtSDH2      DLVEIRLDGINNFQPQKDLQVLLNNNPLPVLIVYR-------------------PIWEGN
SlSDH2      DCVEIRLDAIHNFQPHKHLQLLFKNKPLPILILYR-------------------PIWERN
EgSDH4      DVVEVRLDCIKQFQAHQDLEIILKSKPLPVIIVYR-------------------PKCEGG
Poptr2      DAVEVRLDYINSFQPSQDLETIIRNKPLPVIIVYR-------------------PRWEGG
Poptr3      DVVEVRLDCISKFQPRQDLETIIRNKPLPVIIVYR-------------------PKWEGG
VvSDH2      DLVEVRLDYINNFHPQQDLEIILRNKPLPVMIVYR-------------------PKWEGG
CsSDH1      DVVEIRLDCINNFQPGKDLEIILTKKPLPVLIVYR-------------------PKWAGG
SlSDH3      DLVELCIDDF-TFSDISQLEELLKQRSLPSIVSFR-------------------PKSPIN
EgSDH1      DLVELRVDAM-SFGRVSEVEELIRRRTLPAIVSFR-------------------LNSARA
Poptr4      DLTELRLDSL-SFSHNSEVEKLIKQRTLPSIVSFR-------------------LEPSRI
CsSDH2      DLVELCIDSM-EFSHISEVDKLIQHPTLPAIVSYR-------------------LKSSRK


AtSDH       QYEGDE---NERRDVLRLAMELGADYIDVELQVASEFIKSIDGKKPGKFKVIVSSHNYQ-
NtSDH1      QYAGDE---VSRLDALRVAMELGADYIDVELKAIDEFNTALHGNKSAKCKVIVSSHNYD-
SlSDH1      QYAGDE---KSRLDALRLAMELGADYIDVELKAIGEFNNALHGNKSAKCKLIVSSHNYE-
FvSDH3      QYDGDE---KHRQDILRLAMELGADYIDVEFQVAHEFIDSINGNKPEKLKVIVSSHNYQ-
Poptr1      QYNGDE---KPRLDALRLAMELGADYIDVELKVAHEFNELLRGNKPGKCKLIVSSHNYE-
FvSDH4      QYDGDE---KSRLDALRLAMELGADYIDVELQVAQEFVDFIRDKKPEKFKVIVSSHNYQ-
JrSDH       QYDGDE---KKRLDALRLAMEFGADYIDVELQVACEFNDSIYGRKPENSKVIVSSHNYQ-
VvSDH1      QYEGDE---NSRRDALRLAMELGADYVDIELKVAHEFINSIHGRKPEKFKVIVSSHNYQ-
EgSDH5      QYDGDD---KPRLETLRLAMELGADYIDVELKVASEFNASIQGRKPEKCKVIVSSHNYE-
CsSDH3      QYDGDE---NERVDVLRLAMELGADYIDVELQVAREFNDSIRGKKPEKCKVIVSSHNYQ-
CasSDH2     QYEGDE---NKRQDALRLAMELGADYIDVELQVAQEFNNSISAKKPEKFKVIVSSQNFH-
VvSDH3      QYEGDE---NKRQDALRLAMELGASYIDVELEVAHEFNNSIYGKKPQNFKVIVSSHNFH-
EgSDH3      QYEGDE---SKRQDALRLAMQLGAHYIDVELEVAHDFNSSILGKKPDNFKIIVSSHNFH-
FvSDH1      QYEGDE---TKRQDALRSAMELGANYIDIELEVAHEFNNSIYEKKPDNFKVIVSSHNFH-
FvSDH2      QYEGDE---KKRQEALILAMELGADYIDVELKVANDFYSSIQGKKPERVKIIVSSHNYE-
Poptr5      QYDGDE---NKRQKALQIAMELGADFIDIELKVAQEFYNFIQGKKPEKVKIIVSSHNYE-
CasSDH3     QFDGDE---TRRQAALRQAVELGADYIDIELKVADEFYKLIQGKKPEKVKIIVSSHNYE-
DkSDH       QYDGDE---SRRQATLHQAMELGADYIDIELKVADEFFSSIQERSPKRPKVIVSSHNYE-
EgSDH2      QYEGDD---SRRLDALRLALELGADYVDVELQVAQEFFSSIQGKKPEKAKIIVSSHNYQ-
VvSDH4      QYDGDE---GKRLDALRLAIELGADYIDVELQVAPEFINSIQGKTSGKVKIIVSSHNYQ-
CasSDH1     QYGGDE---NMRLDTLRLAKELGADYIDLELKVASHLMEEHNSNKHRSSKIIVSRYIDG-
NtSDH2      EFEEDDDHIHKQLEVLRWAKELGADYIELDLKIASDFTKKEKPRWSSGCKVIASCFVD--
SlSDH2      DFEADA---HKQLEALRLAKELGADYVELDLKIASEFAKNEKSSWSSGCKLITSCFVD--
EgSDH4      LYEGDE---TARLEALHSALKLGADYVDFELKVAAELMSKQNKLHCGGTKVIVSCFLDG-
Poptr2      QYEGDE---HTRLEALRLAHELGADYIDVELKVASDLVREVKNKHQTGGKVIVSSYLSG-
Poptr3      QYEGDE---HRRLEALRLANDLGADYIDLELKVASELIWELKNKHQNGGKVIVSSYLNG-
VvSDH2      QYEGDE---HSRLEALHLAEKLGADYIDFELKVASDFLGKQKMDQHSSSRTIVSCYVDG-
CsSDH1      LYEGDE---HKRLEALHLAEDLGADYVDFELKVASNILGKQYSSHQSGTRFIVSCNLDC-
SlSDH3      S---EG--KKTCIQVLKLAVELDVEFVEVDTQVVCHQVVAELMKSRSNSKIIASTYVNG-
EgSDH1      SRRQND--KTTCLQVLRLALELDVEFVEMEHEVVSHFNIDELMEKRSSSKIIVSRHLNG-
```

```
Poptr4      SSNKDR--KNTCLQVLRLAFDLNVEFVEMDYEVASEDVMAEYVYNRSNTKLIVSSYVNG-
CsSDH2      SSDEAC--KNTCLQVLRRALDLDVEFVEMDYEVASDPLMSEIIYSRSNTKIIVSSYLNGG

AtSDH       NTPSVEDLDGLVARIQQTGADIVKIATTAVDIADVARMFHITSKAQV---PTIGLVMGER
NtSDH1      NTPSSEELGNLVARIQASGADIVKFATTALDIMDVARVFQITVHSQV---PIIAMVMGEK
SlSDH1      STPSAEDLGNLVARIQASGADIVKFATTAQDITDVARVFQITVHSQV---PIIAMVMGEK
FvSDH3      DTPSVKDLGDLVARIQATGADIVRITTTALDITDVARIIQISVHSHV---PVIGLAMGER
Poptr1      NTPSVEELGNLVARIQAAGADIVKIATTALDISDVARIFQITVHSQVRSVPIIGLVMGER
FvSDH4      ETPSVEALGNLVAAIQATGADIVKIATTALDITDVVRIFQITVHSQV---PIIGLVMGER
JrSDH       DTPSAEDLGNLVARIQATGADIVKIATTALEIADVARIFQITVHSQV---PIIGIVMGER
VvSDH1      NTPSVEDLGNLVVSIQATGADIVKIATTALEITDVARIFQITVHSQV---PVIGLVMGER
EgSDH5      NTPSAEDLSNLVARIQAAGADIVKIATTALDITDVARMFHITVHSQVSSVPVIAMVMGER
CsSDH3      YTPSVEDLSNLVARIQASGADIVKFATTALDITDVARVFQITVHSQV---PIIGLVMGER
CasSDH2     STPSAEAIGNLVARIQATGADIVKIVTTALDITDVARIFQITVHSQI---PMIGIAMGER
VvSDH3      NTPSTEAIGNLVARIQASGADIVKIATTALDITDVARVLQVTVHSQV---PTIAIVMGER
EgSDH3      NTPSAEAIGNLVARIQATGADIAKIATTALDITDCARIFQITVHSQI---PIIGIVMGER
FvSDH1      NTPSSEAIGNLVARIQATGADIVKIATTALDITDCARIFQITVHSQV---PTIGIVMGER
FvSDH2      STPSAEEIGNLVATIQATGADIVKVATTALDITDNASIFQVLARSQV---PMIGLVMGDK
Poptr5      CTPSIEEIGDLVARIQATGADIVKVATTALDITDNARMFHIIVNLQV---PMIGLVMGER
CasSDH3     NTPSAEEIGNLAARIQATGADIVKIATTAQDITDSARIFQLLAHSQV---PTIGIVMGER
DkSDH       NTPSAEEIGNIAARIQATGADIVKIATTALDITDSARILQLIAHSQV---PTIGLAMGER
EgSDH2      NTPSSEELGNLVAKIQATGADIVKIATTALDISDCPRIFEVLAHSQV---PTIAIAMGER
VvSDH4      NTPSAEELGNLVARIQATGADIVKIATTALDITDCARIFQVLAHSQV---PTIGIAMAER
CasSDH1     TTPSEEDLSQLVAHMQSTGADIIKLVSKSSSITELPRFFHLLSHCQI---PLITYSIGDR
NtSDH2      NVTSKEDLSQVVAHMQSTGADILKIVTNANDITELEKMFHLLSHCQV---PLIAYSLGER
SlSDH2      NVTSKEDLSQVVASMQSTGADILKIVINANDITELEKTFHLLSHCQV---PLIAYSVGER
EgSDH4      VTPSKEELSNLATHMQATGADIIKIVTSASNITELARLFHILSYSQM---PVVAYAVGER
Poptr2      ATPSKEDLSHLVASMQATKADIIKVVSNANDITELDRIFHLLSHMQV---PAVAYSLGER
Poptr3      ATPSKENLSHLVATMQATEADIIKVVSNADDITEMERIFHLLSHCEV---PAVAYSVGER
VvSDH2      VTPPIEDLICRVALLQSTGADMIKLVINATNITEITKIFHLLSHCQM---PLIAYSIGDR
CsSDH1      ETPSEEDLGYLVSRMQATGADIIKLVFSVNDITEIARIFQLLSHCQV---PIIAYSVGER
SlSDH3      GNPTKDTLCNSIINLQSTGADIIKLVIDVAYITDVAPVFHMLTHSQV---PLIVRAAGDR
EgSDH1      DKPCKEKLGNLIALMQSSGGDVIKLLVDVDYITDLAPIFQLLTSCQV---PLIATTVGDR
Poptr4      RKPSAEELGYLIACMQSTGADVLKLVLDVEKITDLAPVFTMLTHCQI---PLIALAVGSR
CsSDH2      GKPTTEKLGDVIACMQATGADVMKLEIAVDSITDLAPVFEMLTHCQV---PLIALAVGSR

AtSDH       GLMSRILCSKFGGYLTFGTLDSSKVSAPGQPTIKDLLDLYNFRRIGPDTKVYGIIGKPVS
NtSDH1      GLMSRILCPKFGGYLTFGTLEVGKVSAPGQPTIKDLLNIYNFRQLGPDTRIFGIIGKPVS
SlSDH1      GLMSRILCPKFGGYLTFGTLEVGKVSAPGQPTVEDLLNLYNFRQLGPDTKIFGIIGKPVS
FvSDH3      GLISRILCAKFGGYFTSGTLHSGIVSVPGEPAIKDILDVYNFRLIRPCTKMFGVIGKPIS
Poptr1      GLISRILCAKFGGYLTFGTLESGVVSAPGQPTIKDLLDLYNFRLIGPDTKVFGIIGKPVG
FvSDH4      GLISRILCAKFGGYLTFGTIDSGAVSAPGQPTIKDILNLYNFRQIGPDTKVFGIIGKPVS
JrSDH       GFMSRILCPKFGGFLTFGTIESGIVSAPGQPTMKDLLHLYNLRRIGPDTKVFGIIGKPVH
VvSDH1      GLISRILCPKFSGYLTFGSLEPGIVSAPGQPTIKDLLNLYNFRQLGPDTKVFGVIGKPVS
```

```
EgSDH5     GLISRVLCAKFGGFLTFGTLESGVVSAPGQPTIKDLLDLYNFRSIGPDTKVFGIIGKPVG
CsSDH3     GLISRILCAKFGGFLTFGTLENGIVSAPGQPTIKDLLDLYNFRQMGPDTKVFGIIGKPVG
CasSDH2    GLISRLLSPKFNGYLTYAALEAGAISAPGQPTVKDLLDLYNFRLIRPDTKVYGIIGKPVG
VvSDH3     GLISRLLSPKFGGYLTYGALEAGAISAPGQPTAKDLLDLYNFRLVKPDTKVYGIIGKPVG
EgSDH3     GLISRILSPKFGGYLTYGALEAGAISAPGQPTAKDLLDLYNFRLIRPDTKVYGIIGKPVG
FvSDH1     GLISRLLSPKFGGYLTYGALEAGAISAPGQPTARDLLDLYNFRLIRPDTKIYGIIGKPVG
FvSDH2     GLISRVLSAKYGSFATFGTLVAGEVSAPGQPTVTDLLELYNFRQIGADTKVHGVIGNPIG
Poptr5     GLMSRVLAAKYGAFLTFGSIEAGVVSAPGQPTVKDLLELYNLRQIEADTKVHGVIGNPIG
CasSDH3    GLMSRILCAKFGGFLTFGALESGIVSAPGQSTLKDLLELYNFRQIGPDTKVHGVIGNPIG
DkSDH      GLISRILCAKFGGFLTFGALESGIHSAPGQPTLRDLLDLYNFRQIEPDTKVHGVIGNPIG
EgSDH2     GLISRILAAKFGGYLTFGAIEAGVVSAPGQPSIKDLLDLYNLRQLGPDTKVHGVIGKPIG
VvSDH4     GLISRILSAKFGSYLTFGSLEAGVVSAPGQPTVKDLLDLYNFRQIGPDTKVHGVIGKPIG
CasSDH1    GLISQLLGPKFDGFLIYGSIEGN--PVPGLPTLHNLRQAYGVDYLNAETKVFGLISKTSG
NtSDH2     GLISQLLGPKFGSVLVYGSLDCN--AVPGLPTLGSLRQAYGVDFMDTDTKVFGLISKPVG
SlSDH2     GLISQLLGPKFGSVLLYGSLDGN--AVPGLPSLASLRQAYGVDLMDNDTKVFGLISKPVG
EgSDH4     GLISQLLSPKFGSNLVYGSIEGM--AVPGLPTLESLRKAYKVEHINSDTKVFGLISKPVG
Poptr2     GLISQLLCPKFGGALVYGAMEGN--SIPGLPTLDSLREAYKVENINSDTKVFGLVSKPVS
Poptr3     GLISQLLCPKFGGALVYGSMEGN--SIPGLPTLDSLREAYKVDCINSDTKVFGLVSKPVG
VvSDH2     GFMSQILCRKFGGFLVYGSMEGS--PVAGLPTLESLREAYKVQYINKDTKVFGLISKPVG
CsSDH1     GLVSQLLSPKFNGALVYGSLKGT--PVLGLPTVESLRQTYKVEHINADTKVFGLISKPVG
SlSDH3     GLISQLLGPKYGAFFVCGSLGGK--YTPGLPSLTTIKQVYKLQYVNPDTRIFGVISNPVG
EgSDH1     GLIGQLLGPKFGGFLAYGSLEG---AIPGLPTLTSLRQVYKIEHMNADTKVFGLISNPVG
Poptr4     GLISQLLGPKFGGFLVYGSLSDK--AVPGMPTLLSLRQIYKLEYINADTKVFGLISNPVG
CsSDH2     GLISQLLGPKFGGFLVYGSLGGK--SVPGLPTLVSLKQVYQLEHINPDTKIFGLVSNPVG
```

```
AtSDH      HSKSPIVHNQAFKSVDFNGVYVHLLVDNLVSFLQAYSSSDFA-GFSCTIPHKEAALQCCD
NtSDH1     HSKSPLLYNEAFRSVGFNGVYMPLLVDDVANFFRTYSSLDFA-GSAVTIPHKEAIVDCCD
SlSDH1     HSKSPLLYNESFRSVGFNGVFMHLLVDDIANFFRTYSSLDFA-GFSCTIPHKEAALDCCA
FvSDH3     HSKSPLLYNEGFKSVGFDGVYLHLLVDDIANFLHTYSSTDFS-GFSVGIPHKEAALKCCD
Poptr1     HSKSPVLFNEAFKSVGINGVYVHLLVDDIARFLQTYSSTDFA-GFSCTIPHKEDAAKCCD
FvSDH4     HSKSPILHNEGFKSVGFNGVYVHLLVDDIANFLKTYSSADFA-GFSVTIPHKEAALKCCD
JrSDH      HSKSPILYNEAFKSVCFNGVYIPLLVDDIANFLQTYSSTDFA-GFSCTIPHKEAALKCCD
VvSDH1     HSKSPHLYNEAFKSVGFNGVYVHLLVDDIAKFFHTYSAADFA-GFSCTIPHKEAALKCCD
EgSDH5     HSKSPLLYNQAFKSAGFDGVFLHLLVDDVASFLQTYSSTDFA-GFSCTIPHKEAAVKCCD
CsSDH3     HSKSPILYNEAFKSVGFNGVFVHLLVDDIAKFFQTYSSNDFA-GFSCTIPHKEAAVKCCD
CasSDH2    HSKSPLLFNESFKKVGLNSVYVHLLVDDVKKFFNTYSSVDFA-GFSCTIPHKEVALECMD
VvSDH3     HSKSPLLFNAAFKKVGLNAVYVHLLVDDVEKFFNTYSAPDFISGCSCTIPHKEVAIKCMD
EgSDH3     HSKSPLLFNASYKSVGLNAVYLHFLVDDVEKFFNTYSAVDFASGCSCTIPHKEAALKCMD
FvSDH1     HSKSPLLFNAAFKSVGLNAVYVHFLVDDVEKFFNTYSSVDFS-GCSCTIPHKEAALKCMD
FvSDH2     HSKSPHLYNAAFKANNFNGIYLPLLVDSVANFINTYNSPDWV-GYSYTIPHKEAGFKCCD
Poptr5     HSKSPHLYNAAFKSVGFNGIYLPLLVDSVANYISTYSSPDFA-GYSYTIPHKEDGLKCCD
CasSDH3    HSKSPHLYNTAFRSAGFNGIYLPLLVDSVANFFNTYSSPDFV-GYSYTIPHKEAGPKCCD
DkSDH      HSKSPHLYNTAFKKAAFNGIYLPLLVDNVANFLNTYSSPDFT-GYSYTIPHKMAGLECCD
EgSDH2     HSKSPHLYNAAFRSVNFNGIYLPLLVDNVANFINAYSSPDFV-GYSYTIPHKEDGLRCCD
VvSDH4     HSKSPHLYNSAFKSVGFNGIYLPLLVDSVKNFLATYSSPDFV-GYSYTIPHKEDGLRCCD
```

CasSDH1	PQQRSPSPYPTFRHVGYNGIYVPMLVDDLKEFFSVYSSPDYA-GFSVGFPYKETVTAFCD
NtSDH2	HSKGPILHNPTFRHVGYNGIYVPMFVDDLKEFFRVYSSPDFA-GFSVGIPYKEAVVSFCD
SlSDH2	HSKGPILHNPTFRHVGYNGIYVPMFVDDLKEFFRVYSSPDFA-GFSVGIPYKEAVVSFCD
EgSDH4	HSKGPILHNPTLRHMNFNGLYVPMFVDNLKEFFEVYSTPDFA-GFSVGIPYKEAVIQFCD
Poptr2	HSKGPILHNPAFRHANFNGIYVPMFVDDLKEFFEVYASPDFA-GYSVGFPYKEAVVQFCD
Poptr3	HSKGPLLHNPTLRHVNFNGIYVPMFVDDLKKFFDVYASPDFA-GYSVGFPYKEAVVQFCD
VvSDH2	HSKGPILHNPAFRHVNYNGIYVPMLVDDLKEFFSIYSSPDFA-GFSVGIPYKEAVTGFCD
CsSDH1	HSKGPILHNPTFRHVNYNGIYVPMFVDDLKKFFSTYSSPDFA-GFSVGFPYKEAVMKFCD
SlSDH3	HSKGPLLHNPAFRHTGYNGIYVPLLVDNVKEFFRVFSCNDYA-GFSVGIPHKEAAVRCCD
EgSDH1	HSKGPLLHNPTFRYVGFNGIYVPMLVDNIKEFFKVYTSQDFA-GFSVGIPHKEAAVACCD
Poptr4	HSKGPVLHNPAFRHTGYNGIYVPMQVDDVKEFFRTYTSSDFA-GFSVGIPHKEAAVGCCD
CsSDH2	HSKGPILHNPAFRHTRFNGIYVPMLVDDVKEFFRTYSGTDFA-GFSVGIPHKEPAVACCD

AtSDH	EVDPLAKSIGAVNTILRRKSDGKLLGYNTDCIGSISAIEDGLRSSGDPSSV--PSSSSPL
NtSDH1	ELNPTAKVIGAVNCVVSRL-DGKLFGCNTDYVGAISAIEEALQGSQP--S----MSGSPL
SlSDH1	EIDPTAKAIGAVNCIIRRP-DGKLFGCNTDYIGAISAIEEGLQGSQP--S----ISGSPL
FvSDH3	EVDPIAKSIGAVNCIVRRPTDGKLFGLNTDYFGSISAIEDGLRGSHDKSN----IIGSPL
Poptr1	EVHPVAKSIGAVNCIIRRQNDGKLFGYNTDYVGAISAIEEGLRASQNVSN----TVGSPL
FvSDH4	EVDPVAKSIGAINCIIRRPNDGKLYGLNTDYVGAISAIEDGLQGSLNGSH----VTGSPL
JrSDH	EVDPVAKSIGAVNCIIRRPTDGKLVGYNTDYVGAISAIEDGLRGSHNSSN----TADSPL
VvSDH1	EVSPVAKSIGAVNCIIRRPSDGKLFGYNTDYVGAISAIEDGLRDLHKISS----TSGSPL
EgSDH5	EVDPVAKSIGAVNCIIRRQSDAKLFGYNTDYVGAISAIEDGLRGSQNGNS----AGASPL
CsSDH3	EVDTVAKSIGAVNCIIRRQSDGKLFGYNTDYVGAISAIEDGLRGRLNVSG----GVSSAL
CasSDH2	EIDPIAKKIGAINNIVRRP-DGTLKSYNTDYIGAISAIEDGLRES--NGSS--PATSSPL
VvSDH3	TIDPIARKIGAINNIVRKP-DGKLTAFNTDYIGAIEAIEDGLRES--NGSS--PAVGSPL
EgSDH3	EIDPIAKKIGAINNIVRRP-DGTLTAFNTDYIGAISAIEDGLRGL--NV-V--SPGASPL
FvSDH1	EIDSIAKKIGAINNIVRKP-DGRLVAFNTDYIGSISGIEDELRGM--NGAI--PAGKSPL
FvSDH2	EIDPNALAIGAISCMIRNPTDGKLKGYNVDYLGAIAAIEEGLRGLGLNGSN--NGSGSPL
Poptr5	EVDPIAKEIGAISCMIRRPDDGKLKGYNVDYLGAIAAIEEALGAS--NGA---PASVSPL
CasSDH3	EVDPIAKAIGAISCMVRKPSDGKLIGYNVDYLGAIGAIEEALGGS--NGSS--SGAVSPL
DkSDH	EVDPIAKAIGAISCMIKKPSDGKLIGYNVDYLGAIAAIEEGLGGS--SSAS--NGSVSPL
EgSDH2	EVDPIAKAIGAISQMIRRPTDGKMIGYNVDYLGAIAAIEEALRAS--NGAS--STTTSPL
VvSDH4	EIDPIAQAIGAISCMIRRPADGKLMGYNVDYLGAIAAIEEGLRAS--NGT---TSVGSPL
CasSDH1	EVDPLAQSIGAVNTIIRRHSDGKLVGYNTDCEASITAIEDALKVWGCTNGE--VSLPSPL
NtSDH2	EVDPLAKSIGAVNTIIQRPCDGKLIGYNTDCEASITAIEDALKVNGLTNGA--AFLPSPL
SlSDH2	EVDPLAESIGAVNTIIRRPCDGKLIGYNTDCEASITAIEDALKANG----E--ALVPCSL
EgSDH4	EVHPLAQNIGAVNTIVRRPSDGKLIGYNTDCEASVTAMEDALQECRCINGE--KSLVSPL
Poptr2	EVHPLAKSIGAVNTIIRKPSDGKLIGYNTDCEGSIASIEDALKDQRYINGA---SLNSPL
Poptr3	EVHPLAKCIGAVNTIIRRPCDGKLIGYNTDCEGSITAIEDALRDQKYVNGR---SLNSPL
VvSDH2	ELHPLAQSIGAVNTIMRRPSDGKLIGYNTDCEASITAIEDALRERGLPNGE--APLNSPL
CsSDH1	EVHPLAQAIAAVNTIIRRPSDGKLIGYNTDCEASITAIEDAIKERGYKNGT--ASFGSPL
SlSDH3	EVDPLAKSIGAVNTIIRRPSDGKLIGYNTDCEACVTAIEDALRERQKTNGH--ASNVSPI
EgSDH1	EVHPLAKSIGAVNTIVRRPTDGKLIGYNTDCEASITAIEDALRERHAANGEARAMDASPI
Poptr4	EVHPLAKSIGAVNTIVRRPTDGKLVGYNTDCDASISAIEDALTERRITQKG--VLEASPL
CsSDH2	EVHPLAKSIGAVNTIIRRPIDGKLVGYNTDCESAISAIEDALRERQGINGV--ASHTSPI

```
AtSDH       ASKTVVVIGAGGAGKALAYGAKEKGAKVVIANRTYERALELAEAIGGKALSLTDLDNYHP
NtSDH1      AGKLFVVIGAGGAGKALAYGAKEKGARVVIANRTYERARELADVVGGQALSLDELSNFHP
SlSDH1      AGKLFVVIGAGGAGKAIAYGAKEKGARVVIANRTYERARELAIVVGAEALSLDELSNFHP
FvSDH3      AGRLFVIMGAGGAGKALAYGAKQKGARIIIANRKYDRARKLADEVGGDALPFADLANFHP
Poptr1      AGKLFVVIGAGGAGKALAYGAKEKGARVVIANRTYERAKVLADIIGGDAITLADLENFHP
FvSDH4      AGRLFVVIGAGGAGKALAYGAKQKGARIVIANRTYDRAREIADTIGGEALSISDLDKFHP
JrSDH       AGKLFVVIGAGGAGKALAYGAKEKGARVVIANRTYDRARELADTIGGDALSLADLDNFHP
VvSDH1      AGKLFVVIGAGGAGKALAYGAKEKGARVVIANRTYARARELADAVGGDALSLADLNNFHP
EgSDH5      NGKLFVVIGAGGAGKALGYGAKEKGARVVIANRTYDRARELAETIGGDALSLADLENFHP
CsSDH3      AGKLFVVIGAGGAGKALAYGAKAKGARVVIANRTYDRARELAETVGGHALSLADLENFNP
CasSDH2     AGKLFVVLGAGGAGKSLAYGAQQKGARVVVANRTFERAKELAEKVGGKALTLEEVNDFHP
VvSDH3      AGKLFVVLGAGGAGKSLAYGAKEKGARVVVANRTFERAKDLADKVGGQALTLAEIENFHP
EgSDH3      AGKLFVVLGAGGAGKSLAYGAAQKGARVVVANRTFERAKELADKVGGQAMTLAEVENFHP
FvSDH1      AGKLFVVLGAGGAGKSLVYGAAQKGARVVCANRTYERAKELADKVGGQAMTLEEVENFHP
FvSDH2      AGRLFVVMGAGGAGKALAYGGKQKGARVVVANRSFDKAKILADKVGGEAITLAELENFHP
Poptr5      AGKLFVVMGAGGAGKALAYGAYEKGARVVVANRTYGKAKELASKVGGQAIALAKLKDFHP
CasSDH3     ASKLFVVIGAGGAGKALAYGGKEKGARVVVANRTYEKAKELASKVDGEAITLAELDDFHP
DkSDH       AGRLFVVIGAGGAAKALAYGGKEKGARVVVANRTYEKAKDLARKIGGESMPLTELNDFHP
EgSDH2      AGKLFVVIGAGGAGKALAYGAMEKGARVVVANRTYEKAKELASKVGGQAITLAELENFHP
VvSDH4      AGKLFVVIGAGGAGKALAYGGKEKGARVVVANRTFEKAKELASKVGGEAMTLAELENFHP
CasSDH1     TGKMFVLVGAGGAGRALAFGAKSRGARVVIFDIDFDRAKSLALAVSGEAQPFENLVSFQP
NtSDH2      AGKLFVLVGAGGAGRALAFGAKSRRAEIVIFDIDFDRAKALAAAVSGEALPFENLASFQP
SlSDH2      ARKMFVLVGAGGAGRALAFGAKSRGARIVIFDIDFDRAKALAAAVSGEALPFEKLASFQP
EgSDH4      AGKEFVLVGAGGAGRALAFGAKTRGARIIIFDIDFERAKMLAHAVSGEARPFGDLPYFQP
Poptr2      AGKQFVVVGAGGAGRAIAVGAKSRGARVIIFDIDLDRAKSLAQVVSGEAQHFDSLAHFQP
Poptr3      AGKQFVVVGAGGAGRAIAVGAKSRGARLIIFDIDLERAKSLARAVSGEAQHFESLAHFQP
VvSDH2      TGKQFVLVGAGGAGRALAFGARSRGAQLVIFDLDFDRANSLAHAVSGEVKLYEDVANFQP
CsSDH1      AGRMFVLAGAGGAGRALAFGAKSRGARVVIFDIDFERAKSLASDVMGAARPFEDILNFQP
SlSDH3      AGKLFVLVGAGGAGRAIAFGAKSRGARVVIFNRKYERAKALAAAVSCDALPYEHLNDFCP
EgSDH1      AGKAFVLVGAGGAGRALAFGAKSRGARVFVFNRNFERAKALADAVSGEAIRYEHLDTFRP
Poptr4      SGKTFVLIGAGGAGRALAFGAKSRGARVIIFNRNYERARALAKAVSGEALPYESLDRFRP
CsSDH2      AGKIFVLVGAGGAGRALAFGAKSRGARVIIFNRNYERAKALADAVSGEALHFEYLHEFFP


AtSDH       EDGMVLANTTSMGMQPNVEETPISKDALKHYALVFDAVYTPRITRLLREAEESGAITVSG
NtSDH1      ENDMILANTTSIGMQPKVDDTPIFKEALRYYSLVFDAVYTPKITRLLREAHESGVKIVTG
SlSDH1      ENDMILANTTSIGMQPKVDDTPISKEALKHYSLVFDAVYTPKITRLLREAQESGAKIVTG
FvSDH3      EDGAILANSTSVGMQPNIDETPIPKHALRSYSLVFDAVYNPRMTRLLSEAAESGVRVVCG
Poptr1      EDGMILANTTSIGMQPKVDETPVSKNALRSYSLVFDAVYTPKITRLLREAEESGAKIVTG
FvSDH4      EDGMILANTTSIGMQPKVDETPISKHALRSYTLVFDAVYTPKITRLLREAEESGAIVVSG
JrSDH       EDGMILANSTSIGMQPKVDETPIPKHALRSYSLVFDAVYTPKMTRLLREAEESGAKIVTG
VvSDH1      ENGMILANTTSIGMQPKVDETPISKHALKYYSLVFDAIYTPKITRLLREAQESGATIVTG
EgSDH5      EDGMILANTTSIGMQPKVDETPIPKHALKHYSLVFDAVYTPKITRLLKEAEECGATIVSG
CsSDH3      EDGMILANTTSIGMQPKVDETPIPKHALGHYALVFDAVYTPKITRLLREAEESGATIVSG
```

```
CasSDH2     EEGMILANTTSVGMKPNIDLTPISKEALKHYDLVFDAIYTPKDTRLLREARECGKIIVYG
VvSDH3      EEGMILANTTSVGMKPKINDTPIPKHALKHYSLVFDAIYTPKDTRLLREAKESGKIIVYG
EgSDH3      EEGMVLANTTSVGMKPKIDETPLAKHALKNYCLVFDAIYTPKDTRLLREARETGAVIVYG
FvSDH1      EEGMILANTTSVGMKPNVDDTPISKQALKHYCLVFDAIYTPKETRLLREAKETGAAVVYG
FvSDH2      EDGMVLANTTSVGMKPKTDQTPIPKEALKNYCLVFDAIYTPKWTRLLTEAQESGAAVVFG
Poptr5      EEGMILANTTSVGMKPRIEDTPLAKEALKHYALVFDAIYTPKLTTLLREAQEAGSTIVYG
CasSDH3     EDGMILANTTSVGMKPKTDATPISKKALNRYSLVFDAIYTPKWTRLLQEAQDSGAKVVLG
DkSDH       EDGMILANATSVGMKPNTDATPISKEALSRYSLVFDAIYTPKWTRLLREAKETGAKVVFG
EgSDH2      EDGMVLANTTSVGMKPNVDLTPLPKNALSRYCLVFDAIYTPKLTRLLREAQEVGAIPVYG
VvSDH4      EDGMILANTTSVGMKPNIDNTPLSKKALSRYSLVFDAIYTPKLTRLLREAQESGAIIVYG
CasSDH1     EKGAILANATPLGMHPNTDRIPVAKGTLGDYTVVFDAVYTPRKTTLLKEAEAAGAIIVSG
NtSDH2      EKGAILANATPIGMHPNKDRIPVSEASLKDYVVVFDAVYTPRKTTLLKDAEAAGAITVSG
SlSDH2      EKGAILANATPIGMHPNKDRIPVPEGSLKDYVVVFDAVYTPRRTTLLEDAEAAGALIVSG
EgSDH4      EKGSILANATPIGMHPNKDRIPVSEVPYLK------------------------------
Poptr2      EKGAILANATPIGMHPSTDRIPVAEATLGNYQLVFDAVYTPRKTRLLEDADAAGAITVSG
Poptr3      ENGAILANATPIGMHPSTDRIPAAEETLGNYQLVFDAVYTPRKTRLLKDADAAGAITVSG
VvSDH2      EKGAILANATPVGMHPNTDRIPVAEETLSDYQLVFDSVYTPRKTRLLKEAEAAGAIIVSG
CsSDH1      EKGAILANATPLGMHPNTDRVPVSEETLRDYQLVFDAVYTPRKTRLLKDAEAAGAIIVSG
SlSDH3      EKGMILANASAVGMQPKSDQTPISKEALRSYELVFDAVYTPRNTRLLQEATEVGATVVSG
EgSDH1      EEGMILANASAVGMEPHADKSPVSKGVLGAYELVFDAVYTPRNTRLLQEAAQAGAIAVSG
Poptr4      VNGMILANASAIGMEPNSDQSPVSKEILKACELVFDAVYTPRNTRLLREAKEVGAVVVSG
CsSDH2      EKGMILANASAIGMEPNSDQSPVPKEALKAYELVFDAVYTPRNTRLLREAAEVGATVVSG
```

```
AtSDH       SEMFVRQAYEQFEIFTGLP---APKE------LYWQIMSKY-------------------
NtSDH1      VEMFIGQAYEQYERFTGLA---SSKG------TFQENYGWILRARSLSLFN--AALLVTF
SlSDH1      VEMFIGQAYEQYERFTGLP---APKE------LFKNIMSTY-------------------
FvSDH3      VEMLIGQAYEQFERFTGLP---APKE------LFRKIM-------------------DNC
Poptr1      LEMFIGQAYEQFERFTELP---APKE------LFQKIM-------------------SKY
FvSDH4      SEMFIGQAYEQFERFTGLP---APKE------LFRKVV-------------------EST
JrSDH       LEMFIGQAYEQFERFTGLP---APKE------LFRKVM-------------------ANN
VvSDH1      LEMFIGQAYEQFERFTGLP---APKE------LFKQFI-------------------SNL
EgSDH5      LEMFIGQAYGQYERYTGLP---AP------KELFRKIM-------------------SKY
CsSDH3      LEMFIGQAYEQYERFTGLP---GKMNAPHLYKFFVLLL-------------------YSF
CasSDH2     TEMLIRQGFEQYKNFTGLQ---APEE------LFRELMSRHA-----------------
VvSDH3      TEMLIRQGFEQYKNFTGLP---APEE------LFRELMSKHA-----------------
EgSDH3      TEMLIRQGFEQYKNFTGLP---APEE------LFRTLMEKHA-----------------
FvSDH1      TEMLIRQGFEQYKNFTGLP---APEA------LFRELMEKHA-----------------
FvSDH2      TEMFLNQAFVQVEKFSGIP---ANKQ------LIRDTLARNT-----------------
Poptr5      TEMFINQAFVQFERFTGLP---APKQ------LIRDVLARNT-----------------
CasSDH3     TEMFINQAFVQFERFTGMP---APKE------LIRE-TLAKNT----------------
DkSDH       TEMFLNQAFVQFEKFTGLP---APKD------LIRE-TLARNT----------------
EgSDH2      TEMFINQAFVQFERFTGYP---EQLK------SLIRSSSSCSRIRAHLLGQIPFEFWIVL
VvSDH4      TEMFINQAFVQFERFTGLP---APKE------LI-REVLVRNT----------------
CasSDH1     VEMFLRQAIEQFNLFTGGK---APQE------FMRRTIFANF-----------------
NtSDH2      VEMFLRQAIGQFHLFTRTK---APEE------FMRDIVMAKF-----------------
```

```
SlSDH2      VEMFLRQAIGQFNLFTGSK---APEE------FMRDIVMSKF------------------
EgSDH4      ------------------------------------------------------------
Poptr2      VEMFLKQAIGQFSLFTGRE---APKD------FMREIVLAKF------------------
Poptr3      VEMFLRQAIGQFNLFTGRE---APKD------FMREIVLAKF------------------
VvSDH2      VEMFLRQAIGQFNLFTGGE---APEE------FMREIILSKF------------------
CsSDH1      VEMFLRQAIGQFNLFTGKE---APKE------FMREIVLAKF------------------
SlSDH3      VEMFVRQALGQFKLFTNGL---APVD------FMRRIVYEQF------------------
EgSDH1      MEMFIRQALHQFKLFTSGL---APEE------FMRKLVLEQF------------------
Poptr4      VEMFIRQALGQFRLFTGGLGIILPVV------STHQLRLNDQLTD--------------M
CsSDH2      VEMFIRQALGQFRLFTGGL---APED------FMRKLVLEQF------------------


AtSDH       ------------------------------------------------------------
NtSDH1      PPKSLHSCVIAMVLDSSALPFVLRRN----------------------------------
SlSDH1      ------------------------------------------------------------
FvSDH3      L-----------------------------------------------------------
Poptr1      ------------------------------------------------------------
FvSDH4      S-----------------------------------------------------------
JrSDH       ------------------------------------------------------------
VvSDH1      Q-----------------------------------------------------------
EgSDH5      ------------------------------------------------------------
CsSDH3      NKFHIFTYFLFSFGNFSAEGTISENHGKVLVWSVWSIHYMLLILFSSVIQHEASLFIFFF
CasSDH2     ------------------------------------------------------------
VvSDH3      ------------------------------------------------------------
EgSDH3      ------------------------------------------------------------
FvSDH1      ------------------------------------------------------------
FvSDH2      ------------------------------------------------------------
Poptr5      ------------------------------------------------------------
CasSDH3     ------------------------------------------------------------
DkSDH       ------------------------------------------------------------
EgSDH2      SSVNLFCCNNKNFPPLLEQGLVKSS-----------------------------------
VvSDH4      ------------------------------------------------------------
CasSDH1     ------------------------------------------------------------
NtSDH2      ------------------------------------------------------------
SlSDH2      ------------------------------------------------------------
EgSDH4      ------------------------------------------------------------
Poptr2      ------------------------------------------------------------
Poptr3      ------------------------------------------------------------
VvSDH2      ------------------------------------------------------------
CsSDH1      ------------------------------------------------------------
SlSDH3      ------------------------------------------------------------
EgSDH1      ------------------------------------------------------------
Poptr4      FPQNY-------------------------------------------------------
CsSDH2      ------------------------------------------------------------
```

```
AtSDH      --------------------
NtSDH1     --------------------
SlSDH1     --------------------
FvSDH3     --------------------
Poptr1     --------------------
FvSDH4     --------------------
JrSDH      --------------------
VvSDH1     --------------------
EgSDH5     --------------------
CsSDH3     GQKYKRSTCTSILCMEIKS-
CasSDH2    --------------------
VvSDH3     --------------------
EgSDH3     --------------------
FvSDH1     --------------------
FvSDH2     --------------------
Poptr5     --------------------
CasSDH3    --------------------
DkSDH      --------------------
EgSDH2     --------------------
VvSDH4     --------------------
CasSDH1    --------------------
NtSDH2     --------------------
SlSDH2     --------------------
EgSDH4     --------------------
Poptr2     --------------------
Poptr3     --------------------
VvSDH2     --------------------
CsSDH1     --------------------
SlSDH3     --------------------
EgSDH1     --------------------
Poptr4     --------------------
CsSDH2     --------------------
```
